# Biomedical IoT: Enabling Technologies, Architectural Elements, Challenges, and Future Directions

**DOI:** 10.1109/ACCESS.2022.3159235

**Published:** 2022-03-14

**Authors:** MOHAMMED ALEDHARI, REHMA RAZZAK, BASHEER QOLOMANY, ALA AL-FUQAHA, FAHAD SAEED

**Affiliations:** 1 College of Computing and Software Engineering, Kennesaw State University, Marietta, GA 30060, USA; 2 College of Business and Technology, University of Nebraska at Kearney, Kearney, NE 68849, USA; 3 College of Science and Engineering (CSE), Hamad Bin Khalifa University, Doha, Qatar; 4 School of Computing and Information Sciences, Florida International University, Miami, FL 33199, USA

**Keywords:** Internet of Things (IoT), biomedical IoT, healthcare, wearable technology, biomedical implantations, constrained application protocol (CoAP), implantable biosensors

## Abstract

This paper provides a comprehensive literature review of various technologies and protocols used for medical Internet of Things (IoT) with a thorough examination of current enabling technologies, use cases, applications, and challenges. Despite recent advances, medical IoT is still not considered a routine practice. Due to regulation, ethical, and technological challenges of biomedical hardware, the growth of medical IoT is inhibited. Medical IoT continues to advance in terms of biomedical hardware, and monitoring figures like vital signs, temperature, electrical signals, oxygen levels, cancer indicators, glucose levels, and other bodily levels. In the upcoming years, medical IoT is expected replace old healthcare systems. In comparison to other survey papers on this topic, our paper provides a thorough summary of the most relevant protocols and technologies specifically for medical IoT as well as the challenges. Our paper also contains several proposed frameworks and use cases of medical IoT in hospital settings as well as a comprehensive overview of previous architectures of IoT regarding the strengths and weaknesses. We hope to enable researchers of multiple disciplines, developers, and biomedical engineers to quickly become knowledgeable on how various technologies cooperate and how current frameworks can be modified for new use cases, thus inspiring more growth in medical IoT.

## I. INTRODUCTION

Internet of Things (IoT) is quickly being incorporated into multiple fields, but there are still some industries, such as healthcare, where incorporation of IoT is much slower [1]. Medical IoT envisions a conglomeration of medical devices and people, which rely on wireless communication to enable the exchange of healthcare data, remote patient monitoring, and ultimately a higher quality of life for the patient. Medical IoT not only improves the quality of life, but it can also provide better care services and create more cost-effective systems for hospitals as well. The implementation of medical IoT in healthcare has had a global exponential increase but still has many challenges that need to be solved to increase implementation. These limitations for applying IoT technologies to the healthcare industry include but are not limited to [2]: scalability, mobility, cost, complexity, management, trust, security, and interoperability.

Interoperability and security seem to be the biggest concerns regarding medical IoT [3]–[5]. Medical IoT is comprised of three parts:

- The biomedical device for collecting data
- A way to connect to the Internet
- The software for processing, securing, sending, and displaying data

With these considerations, it is important to ensure the functionality of the devices. Thankfully, there has been a rise in various sectors of medical IoT, such as wearable health devices. Despite being medically themed, the fitness monitors are often used on the commercially driven side of IoT. There also are many limitations to wearable healthcare devices such as the delay in the release of the devices due to government approval and an increased risk to investors. They are scaled down in size and unavoidably have trouble competing with real medical devices [6]. Additionally, strict and burdensome standards have to be established before any human testing can be done (even on prototype devices) [7], [8]. Additionally, the barrier for entry into medical IoT is high due the advanced and interdisciplinary nature of the hardware involved.

Despite these drawbacks, medical IoT has plenty of benefits. Healthcare is now a progressively strained industry, so the need for longer-lasting care will proceed to improve [9]–[11]. Medical IoT can offer:

- home monitoring services that reduce hospital and hospice occupation
- automation benefits and home monitoring services that reduce the cost of healthcare
- health and wellness services that keep the aging population in working condition longer

Research implies patient healthcare costs may be preserved by streamlining and discussing overall health information making use of medical IoT for the management of chronic health conditions [12].

A benefit of medical IoT is efficiency which can solve one of the most challenging problems in the medical field: how to access the correct information in highly dynamic environments. We can guide medical staff and patients on the right course of treatment, thus providing higher patient satisfaction. In short, there are numerous benefits of medical IoT, summarized by [32] and [33]. These benefits include but are not limited to:

- Remote Patient Monitoring: healthcare professionals can remotely monitor patients due to the various sensors that can perform specific tests on the patient’s body. This can benefit those who are chronically ill, and the elderly.
- Cost Reduction: Considering remote patient monitoring, doctor visits and hospital stays will likely be reduced.
- Real-Time Monitoring: Medical IoT devices can collect and transfer patient data such as blood sugar, weight, and other data to doctors. Ideally, constant management and timely intervention can be achieved.
- Geographical Independence: The patient’s data can be available to healthcare professionals regardless of location which can ensure that remote healthcare delivery is still implemented.

Additionally, other benefits include simplicity, affordability, ease of use, and a higher quality of life for patients. Ease of use is a particularly important benefit because patients need to feel comfortable using the medical IoT devices without issue, and medical staff need to know how to operate the IoT medical devices as smoothly as possible [32]. Surprisingly, there has been a lack of research regarding user-experience and usability of medical devices as well. Both user-experience and usability relate to ease of use because comfortability using the medical devices indicates areas that should be improved. Future research should understand that current standards do not always reflect ease of use, which can increase the acceptance of medical IoT devices. [34]

Medical IoT can be easily confused with Healthcare IoT and Biomedical IoT because the terminologies sound very similar. As a result, they are thought to be interchangeable. Unfortunately, many works do not seem to offer a distinction on how to differentiate them. Medical IoT differs from Healthcare IoT which usually refers to procedures, tests, diagnostics, and treatments. Medical IoT and Biomedical IoT are interchangeable since they both refer to tools, hardware, software, and platforms [7]. Throughout this article, we will be using the term medical IoT.

### A. PURPOSE OF STUDY

The purpose of this article is to expand on the current protocols and architectures of medical IoT. While there is research on this topic, sufficient progress has not been made regarding the understanding medical IoT on a deeper, technical level. Despite its promising potential, adoption of medical IoT has been slow. We currently do not have a sufficient understanding of medical IoT and are not able to see the bigger picture of how exactly it benefits healthcare. Specifically, this work will attempt to answer the following questions:

- What architectures have been proposed for medical IoT and what do they attempt to address?
- Which standards are relevant for medical IoT and what shortcomings currently exist within these standards?
- What aspects of medical IoT have yet to be discussed?

### B. CONTRIBUTIONS

The outline of the contributions of this paper in relation to recent literature in the field can be summarized as follows:

- Compared to other survey papers in this topic, this survey provides a deeper understanding of the most relevant protocols and technologies used in medical IoT to enable researchers, developers and engineers to quickly cooperate together without having to struggle with steep learning curves in regard to learning these technologies and standards specifications.
- We provide detailed use cases to illustrate how medical IoT is applied in various medical scenarios and how different protocols presented in the paper fit together to achieve desired goals.
- We focus solely on medical/implanted sensors, with emphasis on their role within the IoT system and why exactly these sensors are called IoT.
- We contribute an overview of some of the key challenges of medical IoT and explain the implications these challenges have on fully integrating it.
- We offer a distinction between Healthcare IoT and Biomedical IoT, since these terms are sometimes used interchangeably with medical IoT, which may confuse future readers.

### C. PAPER ORGANIZATION

The remainder of the paper is organized as follows: There are a total of six sections. Section II provides previous works relating to our paper to provide distinguishing central ideas. Section III discusses enabling technologies of medical IoT. Section IV discusses the most relevant protocols for Medical IoT. Section V discusses the architectures and standards of medical IoT. Section VI outlines applications of the common biosensors for medical IoT. Section VII discusses challenges of medical IoT, and finally, Section VIII concludes the paper with added ideas for future research directions.

## II. RELATED WORKS

Medical IoT has been covered by some other researchers, but literature in medical IoT tends to be scarce, depending on the topics of medical IoT that are covered. Nevertheless, several works do their best to discuss medical IoT or related subjects to it as shown in table 1.

While the works related to this paper are impressive, some discuss issues of medical IoT that have already been covered in other studies. In comparison to other works, we cover a broader range of topics regarding medical IoT. We also cover many use-cases for medical IoT and various architectures that have been proposed for medical IoT as well. Unlike some of the other works, we also discuss current security issues and challenges regarding medical IoT. Additionally, we provide comprehensive detail on specific parts of medical IoT as well, mainly biosensors. These are covered in both use-cases and different types are discussed in detail.

Various aspects of medical IoT in terms of strengths, weaknesses, and prospects are common topics in the field of medical IoT. Integration of medical IoT with cloud computing is an observable topic, and literature analysis shows limitations are still prevalent in medical IoT. The limitations include: computation capability, communication protocols, scalability, infrastructure, and data security [35]. It is important to address the main enablers for medical IoT regarding wearable medical devices [36]. Relevant characteristics for attaining, sustaining, and improving success of wearable medical devices was researched.

Telemedicine topology mapping/architecture pertaining to disease prevention and health promotion presents the following challenges: data transmission and processing, big data, healthcare accessibility/availability, healthcare affordability, IoT device interoperability, ageing population, patient mobility, patient safety, privacy and security, high battery power consumption, network communication [37]. Analyzing new product development, design, and user acceptance is another important research topic pertaining to our topic. Specifically, available IoT home-use devices are undoubtedly beneficial, but future research should identify and address unique risks that are creating gaps in existing research [38]. IoT, Cloud Computing, Fog Computing, and Big Data are changing the healthcare industry [39].Adopting and accepting these technologies can lead to the revolutionary Industry 4.0.

The medical industry is constantly evolving, so research that outlines challenges and future trends of the current enabling technologies is beneficial [40]. Security measures are one of the most important areas of focus regarding new technologies being constructed. It is also important to note that aiding in the development of elderly care can lesson the strain put on the healthcare industry. Regarding disabilities, demands for IoT devices in vulnerable populations (i.e., the elderly) are inconsistent depending on the specific disability [41]. Combining the priorities and individually evaluating patients in a patient-centered environment can effectively determine the implementation of Home IoT.

It is vital to review current articles that provide an overview of healthcare IoT as a whole, and present future directions so that current use cases can be improved. Big data analysis is a recent trend that is becoming popular due to the large sample sizes and continuous data collection for a more global view of research topics [42]. For storing data and delivering information, blockchain is a secure and transparent technique. Fog computing is becoming popular to mitigate the issue of network computational power. For precise sensing, the Internet of Nano THings (IoNT) is recommended. Many people are wary of adopting healthcare IoT but working to eliminate the associated challenges can lead to widespread adoption.

Medical IoT, Artificial Intelligence (AI), edge/cloud computing, security, medical signals fusion, challenges, and future research directions are all topics discussed in our article. Some of the known challenges relating to IoT sensors are interoperability, security, device communication and management, and information management [43].

Ensuring the security of patient information when handling data received by IoT devices is an area of medical IoT that needs to be continuously improved [44]. While protecting information in a controlled environment proved to be possible, global protection possesses challenges due to the deficiency of standards.

Medical IoT has been a revolutionary force due to its usage resulting in lower costs, improved service delivery and higher patient care quality. Despite these benefits, the adoption rate of medical IoT is still very low [45]. There has not been enough research addressing reasons for low adoption of medical IoT. Possible factors may influence adoption and usage of medical IoT using Unified Theory of Acceptance and Use of Technology (UTAUT) combined with Perceived Risk (PR) to evaluate social factors influencing user adoption intention and behavior. These variables predict over 69% of medical IoT system acceptance and usage, but PR was not a significant factor.

Another useful consideration is the identification and mapping of current IoT developments with respect to medicine. A home setting is the most popular place for medical IoT applications [46]. Another noteworthy observation is that neurology, cardiology, and psychiatry/psychology are the medical subfields receiving the most IoT attention. Furthermore, India, China, and the United States are the most involved countries in medical IoT research.

With the event of the recent COVID-19 pandemic, it is important to observe the application of medical IoT for COVID-19. Specifically, different applications of Cognitive Internet of medical Things (CIoMT) are essential [47]. CIoMT is more suitable since people will be connected and monitored via a massive network. Application of medical IoT is extensive regarding COVID-19, since it can provide on-line medical service for patients. The authors conduct a literature survey using the terms “COVID-19” and “Cognitive IoT” or “Corona virus” and “IoMT.” Observing specific diseases will add to the value of medical IoT research as a whole.

Using fog computing, simulation, and experimental proportions in comparison with cloud computing can decrease latency [48]. If a healthcare application is in the process of being constructed in real-time requiring high response times and low latency, fog computing is a recommended method.

Medical IoT in the context of privacy and retrieving medical images is one of the most important conflicts of implementation [49]. Specifically, improving medical image and medical data retrieval using a blockchain-based system with privacy protection is a promising solution. The architecture consists of four layers: the physical layer, transaction layer, service layer, and application layer. Each layer has its own responsibility. The architecture can take advantage of encrypted imaging features used for the medical images. More use cases regarding the incorporation of blockchain and medical IoT would be helpful before this theory can be accepted.

Analyzing medical IoT systems from the human perspective is critical to gain acceptance. Two specific use cases that contain medical IoT components are: Remote Elderly Monitoring (REM) and Smart Ambulance Systems (SAS) [50]. While several research and commercial efforts on designing healthcare systems have been made, the majority of these efforts typically focuses on a system view, (e.g. the interconnection of different subsystems and multiple components, that comprise the system’s architecture), ignoring the human user. Focusing on the human perspective will ultimately lead to greater satisfaction and acceptance from its users, allowing a more widespread adoption of medical IoT. Considering the benefits, vulnerabilities, risks, threats, legal issues, and knowledge gaps that are prevalent in medical IoT are common topics in need of improvement. Suggestions on mitigation strategies that can be implemented to circumvent future security threats should be identified [51]. Potential barriers that prevent medical IoT from being adopted throughout the healthcare industry include:

- Outdated funding, business, and operating models
- Interoperability
- Data breaches
- Adjustment to emerging technologies
- Shortage of relevant digital talent among IT staff
- Patients’ lack of comfortability with regular sharing of their health information
- Scalability

Three main areas for development regarding current and emerging Healthcar IoT technologies include [52]:

- Sensing, where there is a high need for miniaturization and power efficiency
- Communications, where the key aspects are connectivity, standardized protocols, and the wide availability of cloud infrastructure
- Data analytics and inference, where availability of large quantities of data and computational resources is revolutionizing algorithms for health management

It is also important to note that latency tolerance is a crucial factor to consider when designing smart healthcare systems because it can impact designs of medical systems regarding patients in critical condition requiring constant real-time monitoring.

Informing the public of the technologies for current and future use is a popular goal in medical IoT. Hardware and sensors of specific healthcare IoT devices, forms of currently used electronic health methods, and market devices should be relayed to the general population [53]. Reviewing existing architectures of medical IoT are necessary to propose a new and improved framework [54]. The main impediments of medical IoT are security and privacy issues. A proposed three-layered architecture consists of the following: a Things layer, an Intermediate Layer and a Back-end Computing layer. Using multiple databases and incorporating motivation factors could produce improved future results.

Different applications in healthcare IoT including: blood sugar, blood pressure, body temperature, and heart rate are popular monitoring areas [55]. The topology, architecture, and platform of Intelligent Healthcare Network (IoThNet) is used to send and receive medical information for communication purposes between different IoT devices. Medical IoT and cloud computing is another area gaining popularity along with, fog computing, Big Data analysis, and mobile-based applications [56]. A homomorphic encryption system to fully protect user data due to its indiscriminate calculation with a Boolean gate which adds/multiplies every computation on the encrypted data is a benefical incorporation for mobile-based applications involving the aforementioned characteristics.

Recently, contributions have been made that focus on improving medical IoT via the use of various approaches provided by the cyber-physical systems (CPS) community [57]. Medical IoT and biomedical devices still face challenges in terms of reliability, safety, and security. It is important that biomedical devices should also not cause harm to the environment or its user. The authors define CPS as the combination of computational entities designed to construct large, scaled computer-controlled systems. With this approach, potential medical applications can be envisioned and implemented. A medical CPS can provide efficient modeling and design approaches for creating reliable and safe medical devices.

There has been a dramatic increase in the number of IoT/AI articles worldwide since 2008 [58]. More research is needed to develop the following areas: system accuracy, security/privacy protection, data collection, and data management. Improving security and accuracy are the two main areas that are necessary for acceptance of healthcare IoT/AI devices by patients and physicians. Using only peer-reviewed articles, neglecting working research studies, and conference materials can be considered limiting factors to be remedied by including more working research material. Sensor-based physical activity recognition and monitoring (PARM) using healthcare IoT is another research area to be evaluated [59]. The challenges regarding PARM include necessary free-living environment, lifelogging monitoring, scalability, extensibility, cost of devices, and considering different types of physical activity. More research is needed involving this topic to ensure improved usability of PARM applications (fitness tracking/monitoring, remote ambient assisted living, remote health monitoring, disease diagnosis, rehabilitation, emergency alerts, and biomedical sensing) in the real world.

Classification regarding commercially available wearables devices and prototypes for medical IoT is necessary to distinguish the best devices for each condition being monitored [60]. Security issues and power efficiency improvements facing popular wearables are two main challenges that should be improved in future research.

IoT applications, challenges regarding security, and counter measures are important considerations in involving biomedical devices [61]. Specific IoT sensor applications involve monitoring glucose level, ECG, blood pressure, wheelchair management, medication management, and a rehabilitation system. Researchers also discuss cryptographic algorithms for security purposes and concluded that the Rivest-Shamir-Adleman (RSA) model outperforms the Advanced Encryption Standard (AES) and Data Encryption Standard (DES) algorithms. Vulnerabilities exist in security pertaining to electronic health within the cloud [62]. Using a security architecture that is distributed and cloud application layers may help circumvent security issues.

## III. ENABLING TECHNOLOGIES OF MEDICAL IoT

### A. BIOSENSORS

**FIGURE 2.**
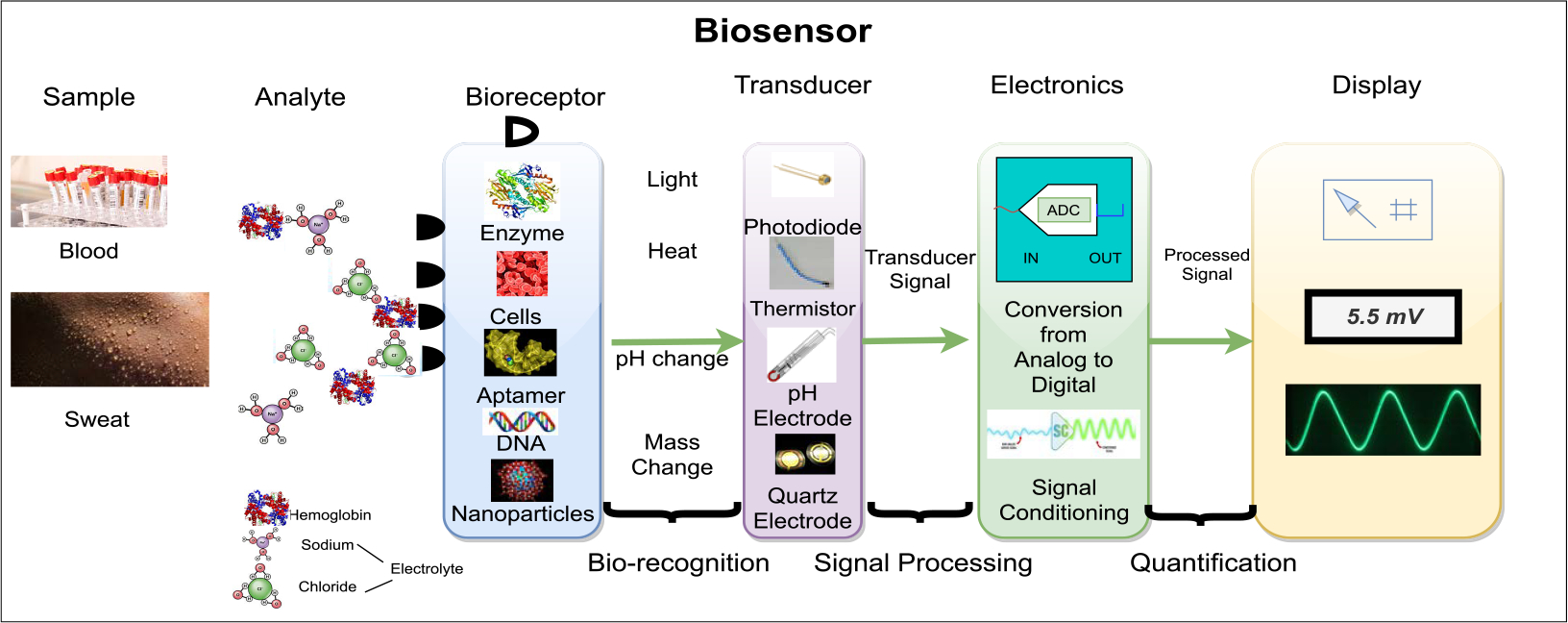
Parts of the biosensor [66].

Biosensors were invented in the 1960s by Clark and Lyons [63]. Near the end of the 20th century is often thought of as the stepping stone for developing biosensors [64]. During the first phase, detecting glucose via electro-chemical detection by making use of the oxidase was the most popular use of biosensors. Within the nineteen-nineties, the interest was occupied with the producing fast, high-sensitive, cheap products just for the natural systems [63]. A biosensor has three components: the recognition material, transducers, and the device for processing signals. These are illustrated in Fig. 2 [65]. In terms of healthcare, they have many use cases, such as diagnostics, imaging, medical therapeutics, monitoring, fitness, and wellness.

Essentially, a biosensor is a kind of analytical gadget usually applied towards measuring any physiological reactions [63]. For a biosensor to be successful it needs to meet the following criteria:

1. Needs to be highly specific and stable under normal conditions.
2. Certain parameters need to be independent to not cause interference.
3. The result has to be precise, reproducible, with no concentration or even dilution.
4. In clinical scenarios, the biosensor must be non-toxic, small enough to fit inside the human body, and ultimately compatible.
5. No device should be inactive for a long-time duration.
6. The sensor should provide rapid measurements of analyzes (from human samples).

Furthermore, there are many types of Biosensors that exist. Each of them have their own unique functions and attempt to complete whatever task is needed [67], [68]. These include but are not limited to: Immunosensors, DNA biosensors and Ingestible biosensors.

Immunosensors are frequently used to detect specific antigens or antibodies. Most of the popular immunosensors are geared toward this [69]. Immunosensors are suitable for medical purposes. Since immunosensors especially bind to pathogens and interact with harmful toxins with the host’s system, they have top awareness and selectivity [70]. The performance of an immunosensor is dependent on three rules:

1. The affinity of the binding components
2. The scale and also ease of access of binding, which should be unchanged after immobilization
3. The density of binding particles coated about the surface area of immunosensor. It should be noted that different strategies can result in different outcomes.

There are different types of immunosensors as well. Materials for each type can vary based on needs [71]. These different immunosensors can include optical, electrochemical, piezoelectric, direct and indirect immunosensors.

Another type of biosensor is DNA Biosensors. These biosensors have been used in fields such as molecular diagnostics, medical diagnosis, and drug-screening [72]. They represented a more effective approach of analyzing the structure of DNA. They are capable of overcoming limits of other sensors thanks to their: fast response time, availability, selectivity, and awareness. In particular, electrochemical DNA biosensors received a lot more interest due to the quick communication time as well as substantial awareness [73]. These sensors have proven to be helpful in detecting anti-cancer compounds, biomolecules, toxins, dopamine, epinephrine and norepinephrine neurotransmitters and even Parkinson’s Disease [74]. DNA Biosensors have good applicability for the medical IoT because of their aid in diagnosing genetically determined diseases and any disease-related to mutated genes or DNA sequence [75]–[77]. Unlike other types of sensors, there is no need for expensive equipment, so this type of sensor can be beneficial to those who are looking for an effective low-cost solution [76], [78].

Furthermore, Ingestible biosensors can be seen as an improved version of wearable biosensors. Ingestibles are a type of electronic devices which is composed of several electronic components for dealing with diagnosis and monitoring of disease. The size of ingestible biosensors can be compared to a capsule that can be swallowed by humans. These types of biosensors need to consider several key aspects, such as size, density, physical structure, and aerodynamics to enable very easy digestion even if the human body is in a state of movement. Furthermore, ingestibles come in many different types, such as imaging capsules, temperature sensing capsules, pressure sensing capsules, and others. Regarding design of ingestible biosensors, they need to be made of biocompatible elements to keep them in working condition while keeping them safe from bodily interaction with the host [79].

The most common usage for ingestible biosensors is for monitoring gastrointestinal health. Typically for gastrointestinal monitoring, it is done via endoscopy, which is invasive. So ingestible biosensors help circumvent this drawback by being as minimally invasive as possible. One example of this comes from [80], where the authors develop ingestible biosensors for gastrointestinal monitoring and diagnosis. Their goal was to detect gastric bleeding. The authors designed a proof-of-concept study, where their proposed ingestible biosensor managed to rapidly diagnose gastric bleeding in a porcine model. One-hundred percent sensitivity and specificity was achieved two hours after device administration. Ingestible biosensors have potential to be used outside of gastrointestinal monitoring; According to [81], there have been usecases of ingestible biosensors being used for areas such as drug discovery and obesity treatment.

Nano-biosensors are also another type of biosensor as well. These types of biosensors are basically sensors which are composed of nanomaterials [82]. Nanomaterials allow for biosensors to achieve a large increase in efficiency and sensitivity, due to their excellent conductivity. Nanotechnology helps make nano biosensors more portable, wearable, and implantable in our body or in any medical device. Nanobiosensors are supposed to detect diseases in early stages at either the molecule level or single-cell level with the help of a biochemical and biophysical signal. They carry high potential in medical IoT [83]. Additionally, there has been recent research interest in implementation and application of them. Recent developments of nano-biosensors have enabled them to be used for detecting diseases such as Covid-19. Nano-biosensors have also been used to detect cancer as well. Unfortunately,nano-biosensors face the challenge of ensuring safety and toxicity regarding nanomaterials, their composition, and particular properties [84].

## IV. MOST RELEVANT PROTOCOLS FOR MEDICAL IoT

For this section, we have categorized these protocols based off the three layers in a typical medical IoT architecture: Perception Layer, Network Layer, and Application Layer. Protocols for each of them will be talked about in the subsequent subsections. Discussion about the Medical IoT Architecture is discussed in section 5.

### A. PERCEPTION LAYER

#### 1) RFID

Objects that utilize radio waves for quick and automatic identification are known as RFIDs (Radio Frequency Identification). RFID has seen first usage in supply chain management, particularly for tracking supplies in a warehouse. The earliest use of RFIDs were in the 1940s to identify airplanes [85], [86]. RFID aims to detect individuals and objects [87].

**FIGURE 3.**
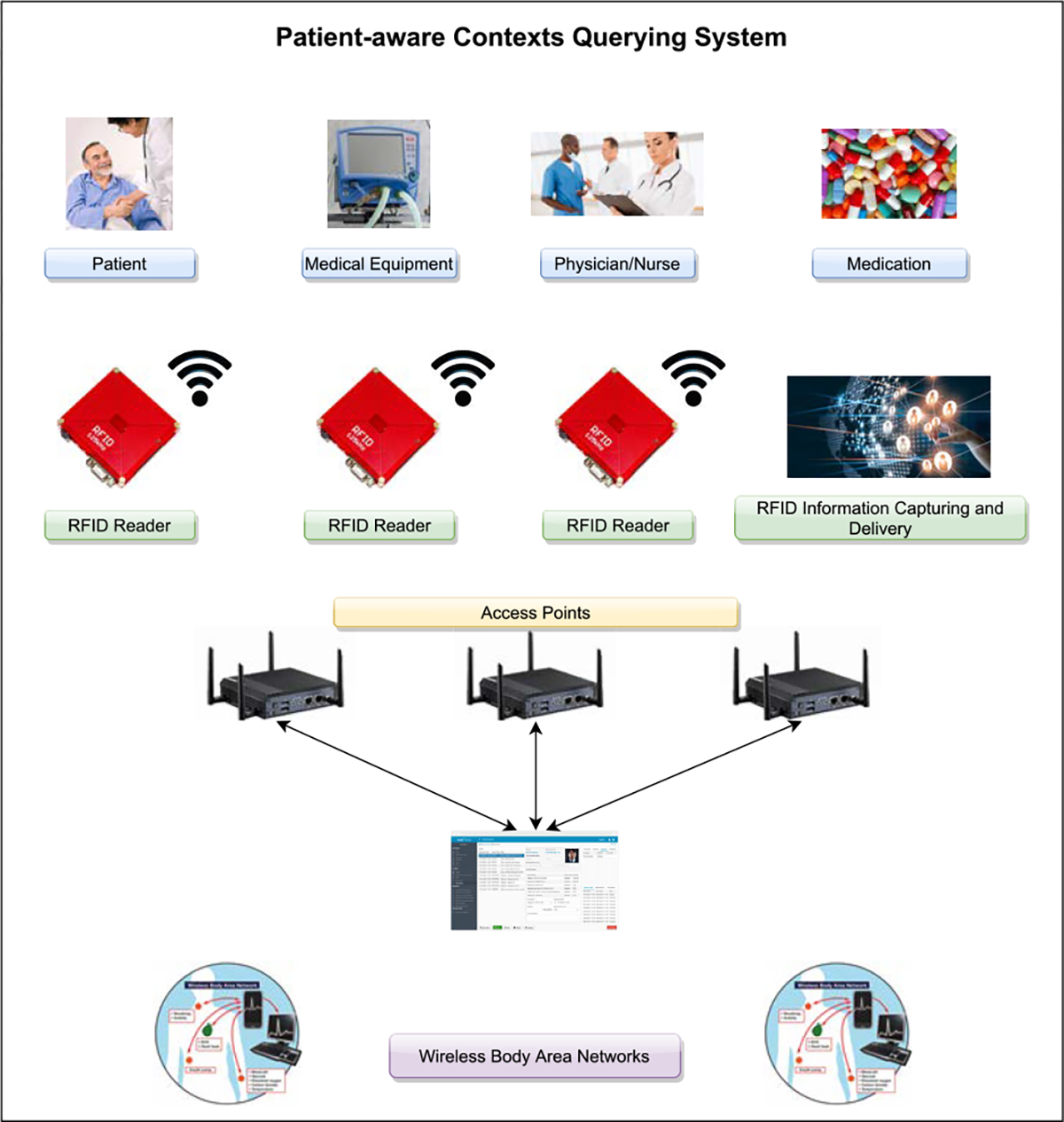
Framework of a health-facility that uses RFID for operations [87].

RFID has enabled medical personnel to spend less time in looking for medical equipment and supplies and more time with their patients. RFID’s application in healthcare is becoming commonplace and have been cited for increasing visibility and efficiency [88]. Outside of healthcare, wearable IoT may come with RFID that can be used by emergency services to access pertinent medical information, but building the architecture is a research challenge [89]. Figure 3 also illustrates a framework of what a health-care facility using RFID would probably look like. In the framework there are a few RFID readers that each have their own access point. These RFID Readers are meant to capture and deliver information regarding patient data and medical equipment. That information gets sent to the medical information system. The WBANS presented in the figure could also be used for sending in patient data to the RFID readers as well. This framework presented may have potential to improve the workflow of a health-care facility by a lot, especially when one considers that RFID has been quite popular in medical IoT.

RFID can be used for a few purposes, mainly tracking, management, and validation [87]. These constructs can assist healthcare in reducing any medical errors, improving productivity, and produce any necessary documentation for administrative purposes [90]. Various findings have shown there is a beneficial relationship between patient safety and RFID usage. However, there are still setbacks RFID needs to overcome for it to be fully implemented in Healthcare. These limitations include obtaining accurate data, cost, and privacy concerns [91], [92]. Despite these setbacks, various countries have started using this technology for a variety of healthcare related tasks as indicated in Table 2. The fact that these countries are using RFID for a variety of healthcare-related tasks also demonstrates how versatile RFID can be. Since a majority of uses for RFID seem to be in patient care, quality of life, and medicine, RFID could possibly be used for other tricky areas of medical IoT such as remote patient monitoring [93]–[95].

#### 2) BLUETOOTH

**FIGURE 4.**
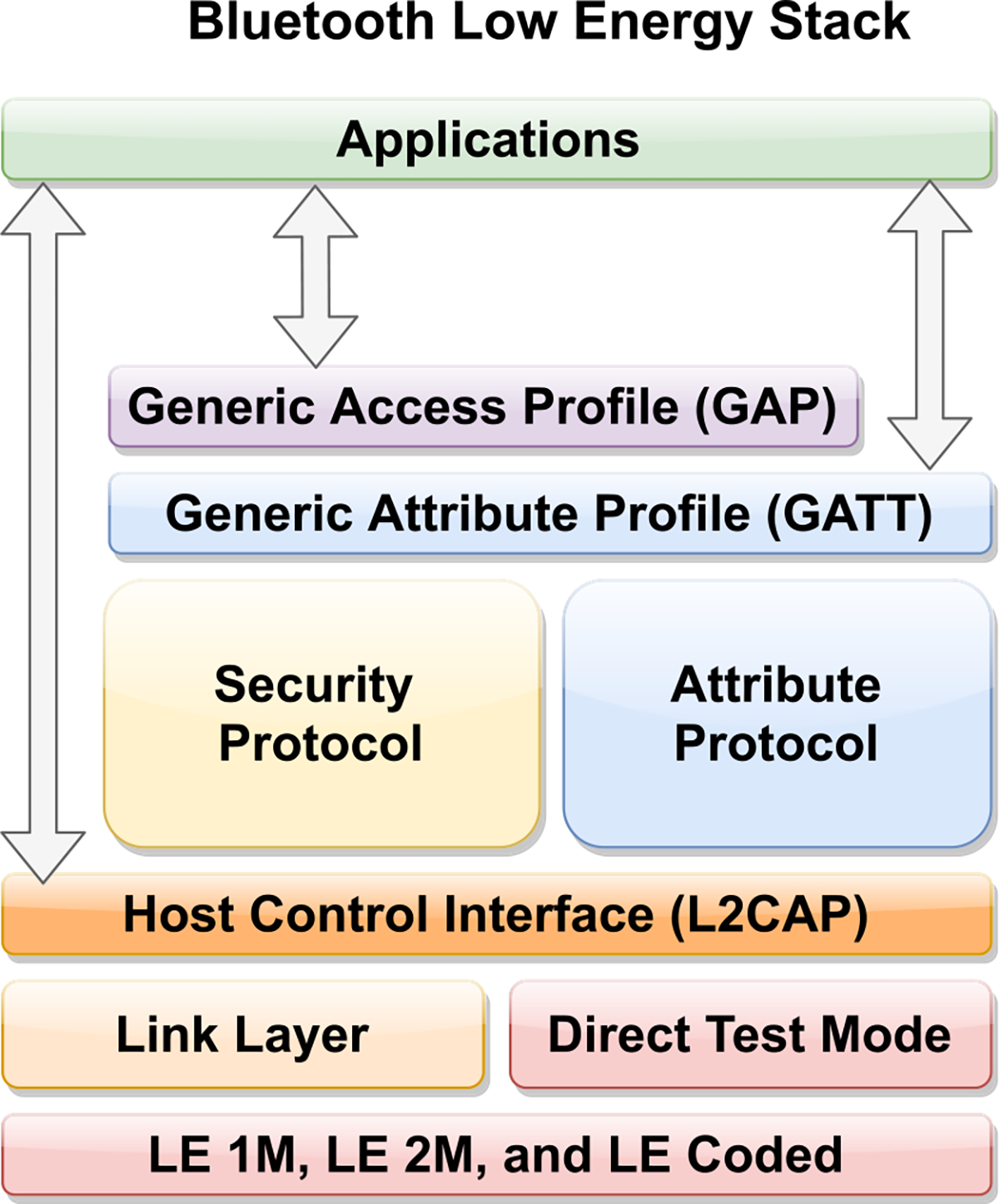
Bluetooth low energy stack protocol.

Bluetooth is a widely used short range communications protocols operating on a 2,402 and 2,480 MHz frequency. Bluetooth is used in devices such as laptops, smartphones, and wearable heath monitors. Mobile operating systems such as iOS and Android also support Bluetooth Low Energy (BLE), making it suitable for healthcare applications [96], [97]. Bluetooth is intended for portable equipment and its applications. One benefit is that Bluetooth devices can connect to the users smartphone [98]. This is especially advantageous for wearable heath monitors, which gain a means of low power connection to the internet without themselves needing the hardware to do so [99]. Figure 4 demonstrates Bluetooth’s framework.

Past releases of Bluetooth have seen large increases in transfer speed: 700 Kbits/s in Bluetooth 1.0, 3 Mbits/s Kbits/s in Bluetooth 2.0, and 24 Mbits/s in Bluetooth 3.0. The focus now has shifted from transfer speed to low power consumption [100]. Bluetooth Low Energy (BLE) has lower energy consumption than traditional Bluetooth, but not meant to transfer a large quantity of data because the limitation of lower bandwidth [101]. Even so, this method could be applied to medical IoT devices that demands higher throughput, real-time, and low-energy transmission. [102], [103].

Benefits of using Bluetooth along with BLE include low power consumption, so battery life can be long if not longer. The longer battery life can increase that battery’s lifetime for a longer duration. As a result, the size of it can be tinier. It also withstands interference and can operate in congested environments thanks to the release of Bluetooth5. In addition, compatibility is not an issue because Bluetooth and BLE are supported by a plethora of devices. Despite BLE’s low bandwidth, it does have a wider communication range [104]. Interestingly, BLE has also been used for Healthcare IOT in terms of blood pressure monitors and fit-bit devices.

#### 3) Z-WAVE

Z-Wave is another technology that has also been used in the healthcare industry [105]. Z-Wave can range up to thirty meters of communication, is mostly for those that just need small data [106]. Z-Wave operates around 900 MHz, making it less prone to inference with other wireless technologies. In addition, Z-Wave also does not need a coordinator and can achieve scalability, with Z-Wave being able to handle up to a certain amount of devices [107]. Z-Wave also has an easier protocol than other technologies, which facilitates easier operation. Z-Wave uses a multitude of affordable hardware, both in power and cost, which can be applicable for IoT. Z-Wave is able to talk with devices via lower-power by using low-power wireless technology. There is reliable data being transferred over to the devices [108]. Regarding its architecture, it consists of a hardware layer, a MACL (Media Access Control Layer), Transport, Routing, and Application layers. All the layers have their own tasks [109].

1. **Physical Layer** - takes care of channel assignments
2. **MACL** - It takes care of network operation based on parameters such as HomeID, NodeID along with other parameters
3. **Transport Layer** - mainly responsible for re-transmission, packet acknowledgment, and others. In this layer there are four basic frame types that are used for transferring commands. These four frame types are: Single-case Frame type, ACK Frame type, Multicast Frame type, and Broadcast Frame type.
4. **Network Layer** - handles updates and routing.
5. **Application Layer**-responsible for decoding and execution of commands.

Benefits of using Z-Wave are that it is suitable for battery-powered devices, thus reducing amount of energy consumed. It also has a strong signal, being able to connect to 100 feet away. Because it is a closed system, it less vulnerable to hackers, so Z-Wave is safer. Unfortunately, because it is a closed system, software patches can only be done by Z-Wave. It is also costly and resource-intensive, meaning that Z-Wave consumes more power, so we would need to replace the battery often. Z-Wave also restricts the number of components and devices connected to it to 232, which may be too small of an amount to cope with [110].

#### 4) MIQoS-RP

This protocol was proposed by [111], and is specifically focused on Wireless Body Sensor Networks (WBSNs). WBSNs are responsible for transmitting time-sensitive and critical data which are obtained from various biosensors. The WBSNs have to consider constraints which include but are not limited to reliability, throughput, and delay. Unfortunately, WBSNs usually have low bandwidth, and high surrounding interference. The proposed protocol was meant to alleviate these problems. The proposed protocol is called the multi-constraint, Intra-BAN, QoS-Aware Routing Protocol (MIQoS-RP). MIQoS-RP’s goal is to minimize end-to-end delay, packet drop ratio and retransmission rate in critical data transmission.

The network architecture of the proposed protocol consists of five small biosensor nodes, one sink node and ten adjacent nodes that are randomly deployed in the network. Each sensor node interacts with other nodes via a multi-hop mesh topology. In a multi-hop topology, a source node interacts with a destination node through multiple routes. The mesh topology provides a peer-to-peer communication session. So, suppose a biosensor node fails, the other nodes of the network continue to work. The protocol also has a routing scheme based on two phases:(1) the initialization phase and (2) MIQoS-aware routing phase. In the initialization phase, the biosensor nodes determine the number of adjacent nodes within their transmission range. The proposed QoS-aware routing scheme extends the routing procedure of a traditional routing protocol. The authors evaluated the protocol’s performance based on terms of average end-to-end delay, throughput and packet drop ratio. The protocol is compared with existing routing protocols as well. Upon evaluating results, the protocol achieved improvements in throughput by 22%, average end-to-end delay by 29% and packet drop ratio performance by 41%.

### B. NETWORK LAYER

#### 1) WSNs

These types of technologies keep track and record some condition, then relay this information to a central hub through a network of spatially dispersed sensors. They are noteworthy for their cost effectiveness and are often battery-powered computing platforms. WSN (wireless sensor network) has traditionally been used for monitoring conditions such as temperature, sound, pollution levels, humidity, and wind, while also controlling critical applications such as home, industrial, military, automotive, and healthcare scenarios [112].

#### 2) 5G

5th Generation (5G) is an emergent advanced wireless system for mobile connectivity, and improves upon its predecessors: 2G, UMTS, 3G, GSM, 4G, LTE, and LTE Advanced Pro. Differences between 5G and LTE are as follows: (1) 5G has a theoretical maximum speed of 20 Gb/s, while LTE is maxed at 1 Gb/s. However, in practice Verizon claims 5G speeds of 300 Mbs to 940 Mb, and AT&T claims 5G speeds of 1.2 Gbs. (2) 5G uses a higher frequency band, resulting in a higher bandwidth and less congestions. (3) 5G towers cover a smaller land area but offer a more consistent coverage. (4) 5G has a reduced latency of 4 milliseconds compared to LTEs 10 milliseconds. 5G can give priority to devices like automated cars and reduce their latency to 1 millisecond [113]–[115].

The development of 5G was a need to cater to future demands of massive industrial and commercial deployment of IoT. 5G anticipates a future where embedded sensors are in virtually everything, enabling machine-type combination like smart cities, smart utilities, agriculture, healthcare IoT, and security alarms. The main take away, as it relates to applications of medical IoT, is that 5G is an ultra-reliable, ultra-consistent, and low-latency wireless system. For example, a moving ambulance will benefit from the consistent and reliable coverage. Transmission of critical high resolution images and video to a central hub within the hospital unit for medical analysis are made more seamless by 5Gs low-latency and high bandwidth [116], [117]. Currently, 5G is being used for implementing wearable compact antennas, which are supposed to improve communication with medical devices. These implementations are discussed in more detail in [118].

#### 3) ZigBee

ZigBee was developed for enhancing WSNs. Zigbee was introduced back in 1998, standards for Zigbee was made in 2003, with revision taking place in 2006 [119], [120]. Benefits of using Zigbee include low price, efficient data, short range of transmission, being easily scalable, reliability, and flexibility.

Zigbee’s architecture consists of a few kinds of devices: a coordinator, router, then lastly an end device. Every system needs to have a coordinator so it can handle information while executing and receiving commands [105]. The routers can transmit data and bring it to other IoT devices. The End devices’ sole purpose is to talk with the nodes in devices. Amounts of routers, coordinators, and end devices in the Zigbee architecture depends on the type of topology. Each topology can vary in terms of how many routers there are [121]. The topologies help devices talk with nodes to handle data the best way possible. Most notably, Mesh is mostly used in comparison to the other two systems cite123. Some of the topologies Zigbee can support are star, mesh and tree [105], [110]. The Zigbee protocol architecture is a stack of layers. Since Zigbee uses IEEE 802.15.4 as its basis, Zigbee is defined by two layers: physical and MAC Layer. The Zigbee protocol architecture has the following layers: The Application, Network, MAC, and Physical layer.

The Physical Layer handles transmitting and receiving signals. The MAC Layer transmits data via different networks [102], [107]. It is also used for security, and there are three types of components for providing protection. The services are: 1) access control, 2) encryption, 3) Integrity. The Network Layer is responsible for handling network management, device operation and disconnections, etc. Meanwhile an APL (Application Layer) is responsible for defining various elements. The APL also contains a sub-layer: the Application sub-layer, which matches devices depending on their requirements in accordance to their services and needs [108].

#### 4) 6LoWPAN

**FIGURE 5.**
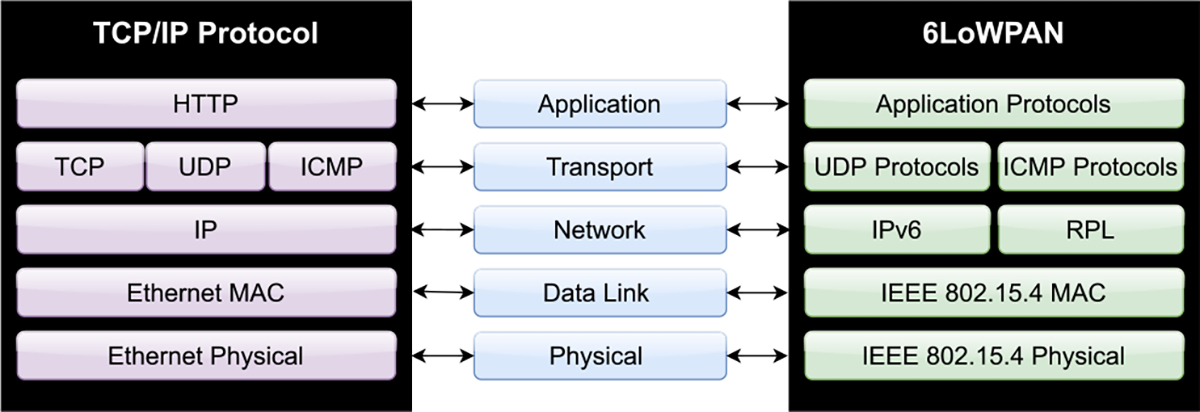
TCP/IP v. 6LoWPAN stack protocol.

6LoWPAN has been utilized in medical care and patient monitoring. Devices using 6LoWPAN can save lives by monitoring the state of a patient to keep track of their health, and react with alarms that can trigger other medical devices in cases where more urgent intervention is required [122], [123]. 6LoWPAN sends and receives packets over networks. 6LoWPAN is distinct from IPv6, and most of the differences pander to optimizations for a miniaturized hardware; These differences are illustrated in Figure 5. Since this is an emergent standard there is some disconformity in standards, but there are groups attempting to standardize a secure and future-proof 6LoWPAN protocol [124]. Benefits of utilizing 6LoWPAN for medical IoT include but are not limited to low power and cost, minimal usage of code and memory, less overhead, suitability for higher density networks, scalability, and ability to communicate with other protocols.

Likewise, when applying 6LoWPAN to IoT, there are several drawbacks of implementing IoT [125]. The layers of the 6LoWPAN can be prone to various attacks [126]. The Physical layer of 6LoWPAN can be prone to jamming and tampering attacks, where the attacker may capture and exploit the node to retrieve information. In its Data Link layer, this layer can be prone to attacks that may drain battery, leading to malfunction and a loss of information. The Network layer undergoes more attacks, so security will be a critical factor in ensuring data is uncompromised [127].

### C. APPLICATION LAYER

#### 1) CoAP

CoAP is an alternative to HTTP (Hyper Text Transfer Protocol) because it can handle constrained environments [112]. It also uses a different format, which is more reliable in terms of space compared to other methods such as plain text. Unlike HTTP, CoAp was built to handle requirements of IoT appliances. The distinguishing factor between CoAP and also HTTP is the transport layer. HTTP relies on the connection-oriented TCP-protocol, while CoAP relies on the UDP protocol, which is connectionless [128].

In CoAP, the client sends requests for an action. Then after the server processes the request, it needs to send a response code. The action that is requested by the client is defined via method and even URL. Another thing to note is that because CoAP uses the UDP Protocol, there is less reliability than one would get with the TCP-protocol [129]. However, CoAP makes up for this shortcoming by using two different levels of messages to provide QoS (Quality of Service): confirmable and non-confirmable. Confirmable messages need to be acknowledged. CoAP allows for negotiation on a preferred representation of a resource, so the clients and servers can evolve independently without effecting each other [130]. The actions defined in the CoAP standard are the following: GET, PUT, POST, and DELETE.

There are different layers that form the CoAP protocol: Messages and Requests/Responses layers. The messages layer uses the UDP protocol to handle messaging. The other layer manages interaction based on the messages. CoAP can support at least four different message types, which are: Non-confirmable, confirmable, reset, and acknowledgement. Confirmable messages are reliable, so the client is able to perform its job properly knowing it is getting reliable information. Should the server have trouble with any interaction, it can send a message back [131]. CoAP has many benefits for usage in IoT. these benefits include:

- **Reduced power requirements and Reduce latency -** Uses the UDP Protocol, and due to this, there is less overhead and it allows for faster wake-up times, so batteries for IoT devices can last longer.
- **A smaller packet size** - Because of CoAP using the UDP protocol, packet sizes can be smaller, which will allow for faster communication
- **Security** - CoAP uses the DTLS (Datagram Transport Layer Security) for securing and encrypting communication.
- **IPv6-based** - Interestingly enough, CoAP was actually designed to support IPv6. As such there may be opportunities for multi-casting.

However, there are several drawbacks to implementing CoAP for IoT one needs to consider which boil down to security and reliability. These drawbacks mainly boil down to security and reliability. Because CoAP uses the UDP Protocol, the UDP Protocol itself does not guarantee delivery or whether the messages have been decoded correctly. Regarding security, even though CoAP does use the DTLS protocol, CoAP is unencrypted by default [131].

#### 2) MQTT (MESSAGE QUEUE TELEMETRY TRANSPORT)

MQTT uses the TCP-protocol and was developed by IBM in 1999 as an interaction between clients and servers. [132] The clients and servers perform different roles. The server is the broker, which is where the clients connect to. The clients themselves connect to the broker so they can perform various operations. Communication takes place through the server, which has the responsibility of authenticating the client to ensure its legitimacy [133]. Since MQTT is not as intensive as the other protocols, it can be used for IoT [134].

More specifically, this particular protocol is actually a publish/subscribe messaging protocol. Here, The MQTT client publishes messages to the server [134]. From there, the message is published to what is called a topic. The clients can follow a multitude of topics and are able to receive whatever messages in each topic. Because MQTT uses the TCP Protocol, the communication between a broker and a client is connection-oriented [135]. This protocol tends to benefit tiny devices with large networks that require monitoring and is also beneficial for networks that struggle with reliability via connections or bandwidth [136]. The session in the protocol is divided into four stages: (1) Connect, (2) Authenticate, (3) Communicate, and (4) Terminate.

The client proceeds by making a connection via default port or either a customized port that is defined by a broker operators [131]. There are two types of codes that are used by these ports: 1) 1883 for non-encrypted communication and 2) 8883 for encrypted communication. Afterwards, the client needs to validate a server certificate and then authenticate the server. However, one thing to note is that since MQTT is designed for constrained devices in IoT, using SSL/TLS may not always be a viable option [132].

One reason MQTT is considered a light-weight protocol for IoT is because of its messages, Messages in MQTT will often be limited to only 256 megabytes. During MQTT’s communication phase, a client can perform the following operations: publishing, subscribing, unsubscribing and pinging. In the publishing task, data gets sent. MQTT can support message sizes of up 256 megabytes, and often the format of the message will be dependent on the application. In case the session needs to be terminated, then a message gets sent to the broker and the connection is closed [137].

MQTT has the benefits of being flexible, meaning it can support diverse application scenarios. Because it is a less intensive protocol, MQTT can guarantee efficient data transmission and quick implementation. Due to the size of MQTT’s data packages, there is less network usage, and small amounts of power can be used. This makes it optimal for IoT. The drawbacks of utilizing the MQTT protocol are that MQTT is not encrypted; This standard did not consider security. Another drawback of utilizing MQTT is its lack of interoperability. This is because the messages in MQTT are binary, so there is no information on how they are authenticated. This may lead to issues in systems where it is required that a variety of systems originating from various suppliers are meant to cooperate together [132], [133], [135].

#### 3) ADVANCED MESSAGE QUEUING PROTOCOL (AMQP)

Interestingly, this protocol was first used in the financial industry [138]. Nevertheless, AMQP has high applicability for broad range of applications, including medical systems. AMQP offers routing and queuing plus reliability and security benefits as well [139]. Specifically, AMQP mandates how clients and messaging providers (also called servers) behave, to ensure interoperability. There are three different components of AMQP: Exchanging, Binding and Message Queuing. These three elements ensure that messages are shared and stored securely and efficiently. Additionally, this protocol is highly useful for exchanging bulk messages [140]. Massive amounts of data can be sent securely to the data store without considering the performance of the whole system. Unfortunately, AMQP is quite vulnerable, being prone to a variety of attacks. These attacks include but are not limited to Denial-of-Service attacks, Replay attacks, masquerading attacks [139].

#### 4) EXTENSIBLE MESSAGING AND PRESENCE PROTOCOL (XMPP)

This protocol is explicitly designed for the IoT environment. The reason is because XMPP allows users to do real time transmission of messages and manage user interaction while being online, offline or busy [138]. Additionally, this protocol has also been used in healthcare as well. XMPP is one of the most extensively used protocols due to its ability to facilitate efficient communication of data between its networks and any involved sensors. XMPP is typically used for low latency and short messages. Furthermore, XMPP is highly scalable because of its non-centralized framework. Unfortunately, there are a few drawbacks to using this protocol. XMPP uses XML format for exchanging data, which is in the form of text. This causes extra communication overhead and leads to delay when transferring data [141]. While XMPP has high security and is scalable, another major drawback is that it has high bandwidth consumption and high CPU/RAM requirements [142].

The discussed protocols each try to improve medical IoT in multiple ways. Unfortunately, many of these protocols have high vulnerabilities that can be easily exploited. For instance, in RFID, the embedded data is unprotected. In Z-Wave, there is no enforcement of a standard key exchange protocol; Additionally, a malicious node can be assigned by the controller, which leaves both the medical data and users vulnerable to malicious hacking. Zigbee’s key transportation for pre-shared keys in the network is insecure. As such, Zigbee also suffers from the drawback of lack of verification. The protocols mentioned in the application layer subsection also suffer performance issues, such as communication overhead. Furthermore, almost none of the protocols address interoperability in medical IoT.

## V. ARCHITECTURES AND STANDARDS OF MEDICAL IoT

The typical medical IoT architecture usually follows the general three-tier IoT framework, which includes the application, network and perception layers. These three layers also have two sub-layers to account for different types of medical equipment, how to transmit data information in an accurate and reliable manner, and patient information plus treatment.

The perception layer is the focal point of medical IoT and is divided into two sub-layers: data acquisition and data access. The data acquisition sub-layer deals with different types of medical equipment and signal acquisition equipment, and collects data information. It utilizes methods such as Radio Frequency Identification (RFID) technology, image recognition technology, and multiple types of sensors. Next, the data access sub-layer uses various access methods for connecting data (that is collected by the data acquisition sublayer) to the network layer. These methods can include ZigBee, Wi-Fi, Bluetooth, and others. The main access methods should be selected according to the environmental characteristics of medical IoT and the needs of different objects [?].

The network layer’s sub-layers are called the network transmission sub-layer and service sub-layer. The network transmission layer uses a mobile communication network and other special networks for transmitting data acquired by the perception layer as accurately and reliably as possible. An important aspect to keep in mind is that its formation was not meant to completely replace the original heterogeneous networks; Medical IoT is about integrating technology of these heterogeneous networks so that they are better suited for hospitals and can improve original networks [143]. Taking this into consideration, the service layer mainly handles the integration of various networks, integrating various data formats, descriptions, and other information. At the same time, it builds a support platform so that the third party can develop relevant applications for the use of medical staff and other relevant personnel [?].

Finally, there is the application layer. The application layer’s two sub-layers are called the medical information application (MIA) and medical information decision-making application (MIDMA) sub-layers respectively. MIA involves medical equipment, patient information management, outpatient information management, inpatient treatment information management, and other elements. Then, the MIDMA layer deals with patient information analysis, disease information analysis, medication information analysis, diagnosis and treatment information analysis [?].

While the traditional medical IoT architecture is comprehensive, it presents challenges. With the traditional medical IoT architecture, it is difficult to account for the connections between large quantities of different medical institutions and information systems, which can lead to isolated frameworks. In turn, this leads to duplicate data being generated, which leads to a huge waste of resources and makes it more challenging to incorporate interoperability between these medical system frameworks [143]. Additionally, the data structure of medical data is unique and complex. There are higher requirements for data storage, processing, and management capabilities. Medical data not only involves the patients’ privacy, but it also affects disease control, decision-making and other aspects. So, the leakage of highly sensitive data will have a serious impact on individuals, families and even society. Therefore, it is difficult for traditional IoMT to ensure the security, privacy, and integrity of massive data [?].

### A SURVEY OF DIFFERENT ARCHITECTURES PROPOSED FOR MEDICAL IoT

A.

Throughout medical IoT’s inception, numerous architectures have been proposed and implemented to further improve medical IoT’s applicability. Some of these architectures are geared toward specific use cases while others attempt to solve current issues in medical IoT. In total, we discuss seven proposed architectures, and a brief overview of these architectures are shown in table 3. Table 3 discusses these architectures in terms of their area(s) of focus and benefit(s).

#### 1) EDGE COMPUTING ARCHITECTURE FOR MEDICAL IoT

**FIGURE 6.**
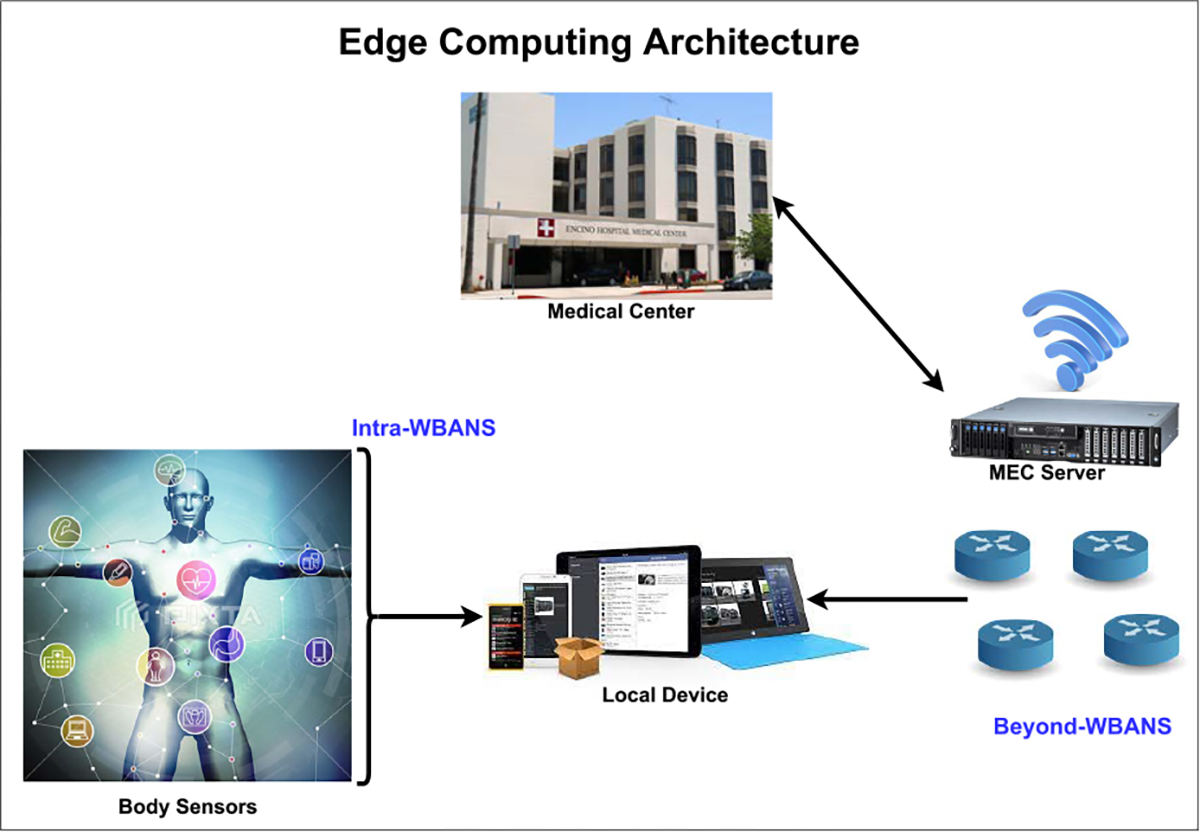
Edge computing architecture for medical IoT [144]. It contains five major components: patients, local devices, body sensors, servers, and the medical center. For the intra-WBANS, body sensors equipped by patients monitor raw health data and transmit that data to the local devices. Beyond-WBANS are primarily concerned with computational resources.

The work [144] proposes a framework that uses mobile-edge computing and also includes 5G. The authors designed the framework for the purpose of home monitoring and minimizing system-wide costs associated with medical IoT. Because the proposed framework uses 5G, the framework can effectively take advantage of 5G’s benefits, such as efficiency and high-speed transmission. The proposed architecture also includes WBANS (Wireless Body Area Networks) as well. The body sensors for WBANS collect different types of health-related packets from living tissue and transmit them to the edge computing service for medical analysis. The authors’ proposed architecture is shown in Figure 6. The authors manage to evaluate their proposed architecture, and their evaluation results indicated the framework was effective in reducing system-wide cost.

#### 2) MeDIC

The authors of [145] propose a framework called MeDIC (Medical Data Interoperability through Collaboration of healthcare devices), which heavily focuses on applying interoperability and is built with the intention of resolving potential data format conflicts between devices. The authors’ proposed framework consists of four major components: Authentication, Publish/Subscribe, Probe, and Translation. The authentication component provides an authentication server on the cloud that maintains a list of patients’ devices with their access credentials and relevant data permissions. Subscribe/Publish part of the framework is supposed to retrieve data after a successful authentication is complete while the resource manager part is supposed to provide data interoperability. Finally, the Probe component of the framework maintains a table that enlists the capabilities and state of available medical devices. In the table, medical devices’ Load Factor is considered. Load Factor refers to the available computational resources for the medical device. Interestingly, the authors evaluated their framework via an IoT simulator that reproduces a smart city. Upon evaluation, the authors’ framework successfully reduces the network traffic and improves the response time.

#### 3) BLOCKCHAIN BASED SMART CONTRACTS FOR INTERNET OF MEDICAL THINGS IN E-HEALTHCARE

In [146], the authors propose a novel architecture for medical IoT that incorporates blockchain and smart contracts. Blockchain is a concept that enables the execution of transactions between two or more parties in a trustworthy manner without the need for any validating or trust authority in between. Blockchain works in a decentralized approach where a copy of data is found at every node, so any new node can update itself from the network. Meanwhile, smart contracts are scripts or series of code that run over the Blockchain for execution. By incorporating these into the blockchain, smart contracts can be executed quickly and securely.

The authors implemented and simulated their proposed architecture using MATLAB Simulink. In the simulation environment, the authors incorporated each node with a block and each block ensures the trust between those blocks. For testing, the authors added compromised blocks. Additionally, the authors also used a dataset with information traffic size of 1024 Bits. Then, to perform malicious activities, the authors mentioned that each set of blocks has been added with a set of malicious nodes and malicious miners. Upon testing, the authors noted that their proposed architecture was highly efficient, achieved low latency, and was very effective in increasing average packet delivery ratio.

What is notable about this architecture is the effective incorporation of smart contracts into blockchain. By adding smart contracts into the blockchain framework, higher security can be achieved. Combining both smart contracts and blockchain together also allows for more efficient data management, which is highly relevant in medical IoT because huge amounts of data are being generated and processing of that data is usually limited [147]. However, there are some potential drawbacks of the proposed approach, particularly with smart contracts which include consistency, immutability, transaction processing speed, and scalability.

#### 4) ON A SECURITY-ORIENTED DESIGN FRAMEWORK FOR MEDICAL IoT DEVICES: THE HARDWARE SECURITY PERSPECTIVE

Here, Nomikos *et al.* [148] propose a framework to address security for medical IoT from a hardware perspective. This is quite notable since security is not greatly emphasized with medical IoT, leading to many wearable devices having low security. According to the authors, the proposed architecture is needed due to hardware attacks posing a serious threat. These threats can either decrease or cancel security levels of these medical IoT devices. Another reason for the proposed framework is to help designers of the devices, embedded system designers, and hardware security experts address other restrictions for medical IoT devices. These restrictions include but are not limited to low-power operation, short time-to-market as well as low engineering/production costs.

**FIGURE 7.**
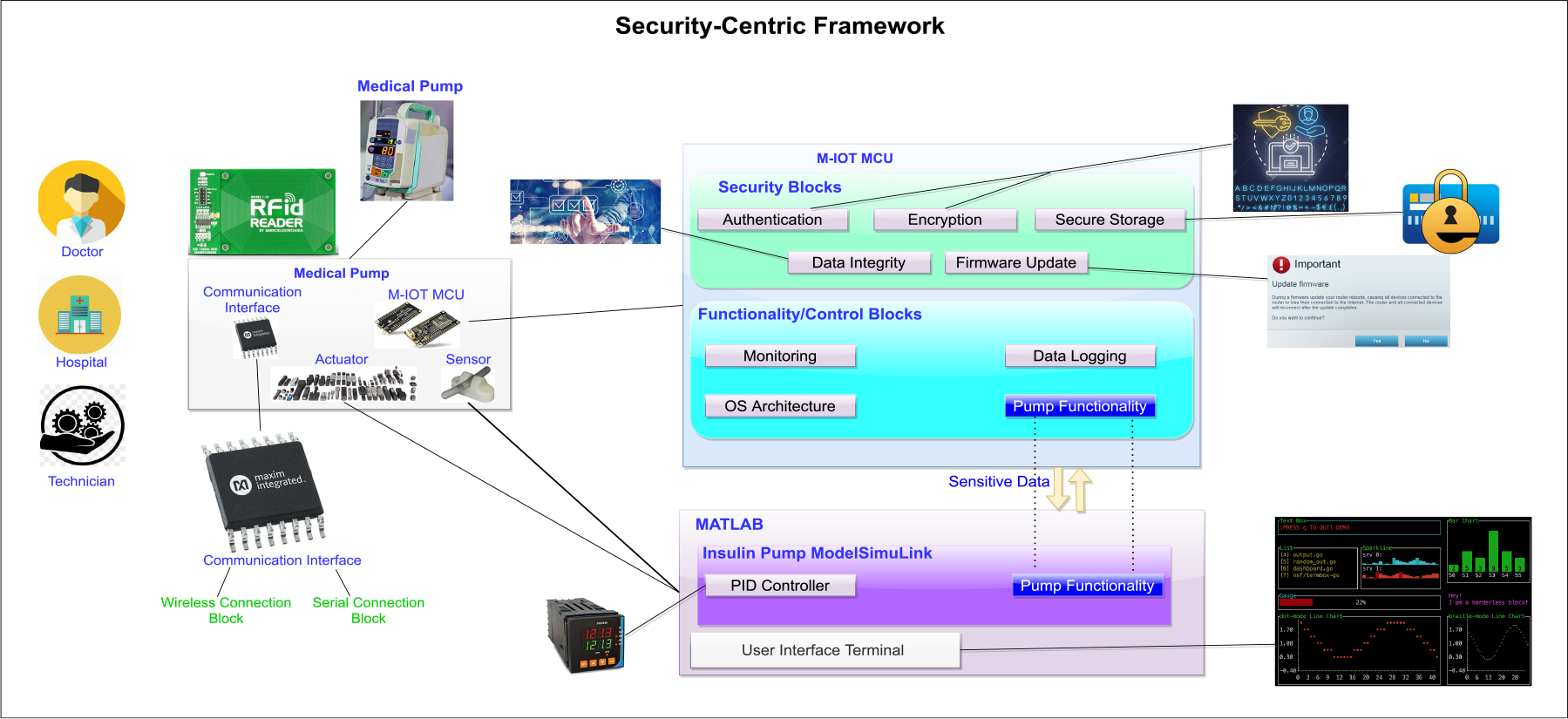
Security-centric framework. This framework uses a medical pump as an example of security from a hardware point of view [148].

The proposed architecture is divided into two distinct domains, as demonstrated in Figure 7. In one domain, there is a medical IoT device including all the supporting circuits. In the second domain, the proposed architecture integrates a higher-level model that runs on a PC. This is supposed to emulate operation of various sensors of medical IoT devices. The authors use a MatLab model of a medical pump. The MCU is the core of the medical IoT device and integrates the main software components. Dividing the MCU into security functionality/control modules enables us to support modularity, thus reducing overall complexity. The security module consists of authentication, encryption, storage, data integrity and firmware updates. The functionality/control module deals with data logging, monitoring, OS architecture, and pump functionality. The communication interface consists of wireless connection and serial connection blocks. The communication interface also focuses on communication protocols with low power consumption. The pump functionality is partially implemented on the MCU and partially inside the high-level model. A benefit of this setup is the ability to isolate sensitive data. To test their proposed architecture, the authors implemented a Side Channel Analysis attack. From there, the authors determined which parts of the architecture were most vulnerable.

Advantages of this architecture are that it allows consistent security validation at design times and considers embedded system design constraints. Furthermore, the architecture is highly adaptable as reusability is achieved. It would have been beneficial for the authors to evaluate their architecture against other types of hardware attacks to further support the architecture’s adaptability.

#### 5) A BLUETOOTH-BASED ARCHITECTURE FOR CONTACT TRACING IN HEALTHCARE FACILITIES

In [149], the authors propose and implement a Bluetooth-based framework for contact tracing healthcare facilities. This framework is geared towards the recent COVID-19 pandemic. The primary targets for this architecture are nursing homes and hospitals, as they have been greatly exposed to the pandemic. Thus, the authors hope that the proposed approach will help investigate ways of building an architecture with various devices to aid in contact-tracing.

**FIGURE 8.**
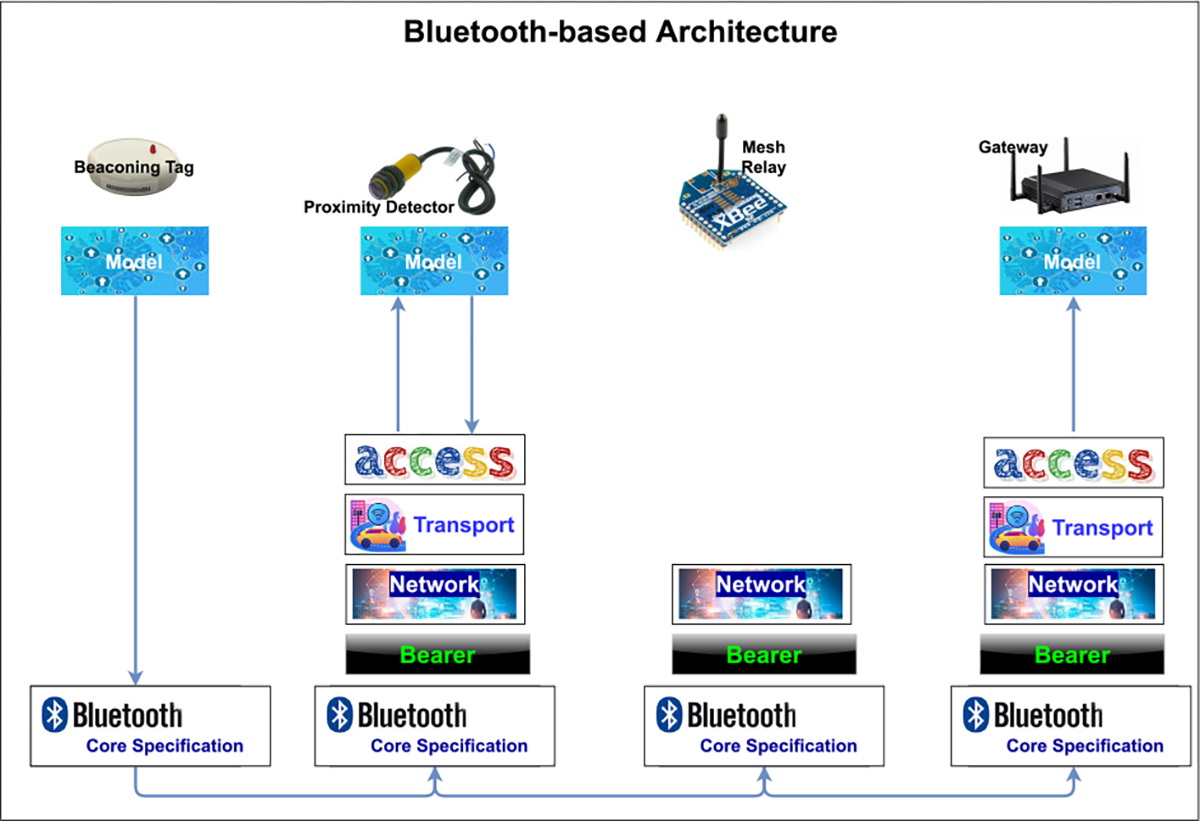
Bluetooth-based architecture [149].

The authors define four different roles of the devices in the architecture, depending on the functionality and resource capabilities, as shown in Figure 8. The involved components are: beaconing tag, proximity detector, mesh relay, and a gateway. The proximity detector is meant to monitor and send messages based on presence, absence or change of position of the beaconing tag. The mesh relay is a node that forwards messages from other nodes. The gateway is a generic mesh node which provides an interface with a contract-tracing server. There, computation of proximity data is performed, and relevant data is stored.

The main benefit of the proposed architecture according to the authors is that the privacy aspects and implication are easier to handle in restricted scenarios for contact tracing compared to other scenarios, since access and the presence of staff and patients are already monitored, and visitors may need to register to access the premises.

#### 6) AN ENERGY-EFFICIENT FOG-TO-CLOUD INTERNET OF MEDICAL THINGS ARCHITECTURE

The article [150] a fog-to-cloud medical IoT architecture to optimize energy consumption. The main argument is that typical medical IoT architectures that incorporate cloud-based systems create bottlenecks, which can cause entire processes to slow down. The proposed framework also utilizes Bluetooth enabled biosensors. According to the authors, the proposed architecture works in three different nodes, and the data is intended to transmit to fog and cloud devices for further processing.

There has been an emerging trend in deploying fog computing for medical IoT, with notable examples coming from [151] and [152]. In comparison to traditional cloud computing and edge computing, fog computing can offer greater privacy control, increased business productivity and agility, and better data security for medical IoT. It should be noted that while fog computing, edge computing, and cloud computing are similar, there are some differentiating factors. The primary difference is the location where data processing occurs. In cloud computing, data is processed on a central cloud server, which is usually located far away from the source of information. Edge computing usually occurs directly on the devices to which the sensors are connected or a gateway device that is in the proximity of the sensors. Contrarily, in Fog computing, the data is processed within a fog node or IoT gateway. Another difference is purpose. Cloud computing is best for long term in-depth analysis of data. Edge and fog computing are more suitable for the quick analysis required for real-time responses. Another distinction is that Fog computing does not require constant online access, so it combines online and offline access.

The framework consists of biosensors, bio-gateways, bio-fog devices, and a back-end bio-cloud system. These devices are reserved for the medical purpose, therefore, called biodevices. The biosensors monitor the body and send data to a bio-gateway where it extracts the essential data through data abstraction. The biosensors and bio-gateway are connected through Bluetooth technology. A bio-gateway connects to a bio-fog through WiFi where it receives the extracted data and transmits to the bio-fog for further analysis and processing. A bio-fog is further connected to the bio-cloud through WiFi, where a bio-cloud provides a user application platform and other services like storage, analysis, evaluation, notifications, and reports. End users, for example, medical experts can get the real-time data and medical history from a bio-cloud. Biofogs are then positioned in different areas to facilitate the local bio-clusters, which helps to reduce response time and increase efficiency of resource allocation.

To evaluate the proposed architecture, the authors use simulation and compare against other existing techniques with respect to delay, throughput, and energy consumption. Results revealed that more power of biosensors was saved, which prolongs the life of the entire network. Additionally, the proposed framework reduced energy consumption between 30 to 40 %.

#### 7) LoRa-BASED MEDICAL IoT ARCHITECTURE AND SYSTEM TESTBED

The authors of [153] propose a new medical IoT architecture that is based on LoRA technology, focusing on homecare and hospital services. The main purpose of this architecture is to improve infrastructures used in hospital and homecare. Many hospitals do not have connected medical devices or applications. Additionally, in homecare applications, Wi-Fi is used, but with Wi-Fi usage there is considerable power consumption, and it only responds partially to the mobility of the user. Also, if the user is not in range of the Wi-Fi connection, medical data is not transmitted, and monitoring cannot be done. The authors designed this architecture to be of low complexity. Edge Computing is also used to provide the proposed architecture with security, and to provide more bandwidth. Specifically, the architecture uses Edge Computing Components (ECCs) to validate acquired sensor data.

Unfortunately, LoRa does have a few limitations. LoRa has limited bandwidth and throughput, so utilizing Edge Computing and other compression algorithms is necessary to transmit higher volumes of data. Additionally, LoRa is affected by interferences with other communication technologies.

### B. RELEVANT STANDARDS FOR MEDICAL IoT

Here, we cover some standards that are relevant for medical IoT. A summary of these standards is described in table 4 below. The table provides a brief overview of the discussed standards regarding the advantages and drawbacks. The standards are further elaborated on in the following subsection.

#### 1) IEEE 802.15.6

This standard is a radio communication framework that can handle a plethora of tasks. It is able to assist in diagnosing and regulating patients including the elderly [154]. Interestingly, it is also popular in medical IoT, specifically for WBANS due to the protocol’s ability to handle heterogenous network traffic, provides high quality of service, and extremely low power consumption [155]. The goal of IEEE 802.15.6 was to implement a way for lower-powered devices to achieve specific medical goals. The framework protocol defines and supports specific layers [156].

The First layer, which is the Narrow Band, turns the transceiver on and off. The second layer operates in different bands that become further divided based on how much bandwidth they possess [157], [158]. The Ultra-wideband (UWB) and Human Body Communications (HBC) layers needs to support one of the channels. The HBC operates in two bands that are centered at different MHz with a certain amount of bandwidth. HBC is responsible for WBAN’s entire protocol [159].

Regarding security aspects, IEEE 802.15.6 can handle at least a few levels of security, each with their own functions, defined below.

1. **Insecure intercommunication Protocol** - This represents the lowest protocol. Here, information is transferred but it’s not secure. This level does not have any rules regarding methods of security.
2. **Authenticating** – This is the middle level of protection. Here, information is authenticated but there is not any additional level of security.
3. **Authentication and Encryption** - Highest level for security, In this last level, there is both authentication and encryption, making this level more secure in comparison to the other two.

#### 2) IEEE 802.15.4

This standard was focused on devices that require a long battery life [159]. This protocol also supports communication that costs less and has a shorter range [132]. The set up in this standard can make the connection more accessible and applicable for IoT. This enables us with managing packets and handling network loss, if any. The protocol is also able to reduce overhead and reduce memory requirements for better management, making IEEE 802.15.4 another fairly reliable protocol [160], [161].

An important consideration is the vulnerabilities in the IEEE 802.15.4 protocol [162]. These vulnerabilities can fall into management and protection. It can be easy to implement this protocol in a way that provides less security than one would expect [110], [163]. Additionally, IEEE 802.15.4 may not be suited for facilitating communication among many IoT devices or for covering large areas. This is relatively crucial for medical IoT, especially for hospitals or other large medical facilities [164]. Thankfully, there has been more focus in recent years for improving reliability, robustness and latency of IEEE 802.15.4, so that it can be more optimized for future medical IoT devices [165].

#### 3) FAST HEALTHCARE INTEROPERABILITY RESOURCES (FHIR)

This standard was created by Health Level Seven International (HL7). HL7 is an accredited organization focused on providing a comprehensive framework and optimal standards for exchanging, integrating, sharing, and retrieving electronic health information. The ‘Level Seven’ refers to the seventh level of the seven-layer communications model for Open Systems Interconnection (OSI) - the application level. The application level interfaces directly to and performs common application services for application processes. The standards developed by HL7 define how information is packaged and communicated from one entity to another, thereby establishing the language, structure, and data types necessary for seamless integration between systems. FHIR is the latest standard published by HL7.

FHIR is a standard that deals with communication of healthcare data and is used for electronically exchanging healthcare information. FHIR was developed around 2012 in response to market needs for faster, easier, and better approaches for exchanging the rapidly growing amount health data. The basic component of FHIR is a resource, which has the following characteristics: (1) A human readable part, (2) a common set of metadata, and (3) A common way to define and represent them, building them from data types that define common reusable patterns of elements. The main idea of FHIR is to create a basic set of resources that individually or in combination, that can satisfy most use cases in terms of healthcare data.

FHIR is unique because it is based on internet standards that are used by industries outside of healthcare. By adopting existing standards and technologies already familiar to software developers, FHIR lowers barriers of entry for new developers to support healthcare requirements. FHIR also provides many advantages as well, which include but are not limited to:

1. Strong focus on implementation
2. Multiple implementation libraries
3. Interoperability - base resources can be used as is, but can also be adapted as needed
4. Concise and easily understood specifications
5. Support for RESTful architectures, seamless exchange of information using messages or documents, and service-based architectures

While FHIR is unique in attempting to handle interoperability, there are still a few drawbacks. Interoperability is the biggest drawback, and unfortunately no technical standard can truly solve interoperability because its barriers are not technological. The barriers for interoperability are related to business and culture. Furthermore, up until recently there has not been enough incentive and desire to create demand for interoperability. Another disadvantage is backwards compatibility, though this will likely be addressed in future versions of FHIR.

#### 4) OpenEHR

OpenEHR is an open standard which supports definition, persistence, management, storage, retrieval, and exchange of structured clinical data in electronic health records (EHRs). OpenEHR is maintained by the OpenEHR foundation based in London. OpenEHR is defined by a three-level modeling approach which consists of (1) a reference model, (2) archetypes, and (3) templates. The Reference model contains definitions of data structures, types, security aspects, and an identifier that helps organize each reference model. The Archetypes define specific concepts related to the healthcare domain and are maintained by physicians (e.g. blood pressure observation). Meanwhile, the templates combine and constrain elements from multiple archetypes to support a given use case. Templates are also utilized by developers for generating various artefacts which can be used to implement applications.

Using OpenEHR has many benefits, one of them being encouraging global standardization, while still supporting local variation. OpenEHR is also designed for clinicians so they can build clinical models. In conjunction with this, OpenEHR’s methodology is highly scalable to cover all of health and social care. Regarding security, it promotes the sharing of open data since the models are separated in such a way the clinical data enables no identification of the patient that generated them.

Important considerations involving standards are the focus and complexity, because every standard has different areas of importance. For instance, FHIR focuses on the exchange of data through provision of simple and easy to use REST API’s while OpenEHR is optimized to provide a data platform, focusing on persistence of data. Additionally, OpenEHR uses over 300 more complex archetypes which brings a higher level of complexity.

#### 5) ISO 13485:2016

ISO 13485 was first published in 1996 and revised, most recently for the third time, in 2016. ISO 13485:2016 specifies requirements for a QMS (Quality Management System) where an organization must demonstrate its ability to provide medical devices and other related services that can consistently meet customer and regulatory requirements.

With regards to the QMS, the standard outlines details in terms of general and documentation requirements, specifically in Section 4. The general requirements dictate that whatever requirement, procedure, or activity is needed; the organization needs to implement them for their QMS in accordance with this standard. The organization is also responsible for determining what processes are needed for the QMS. Furthermore, the organization needs to determine relevant criteria and methods to ensure the processes are operating effectively and monitoring these processes. For the QMS, the organization is also responsible for ensuring that resources are available to support operation. Finally, the organization is responsible for validating computer software used for the QMS, and the software applications need to be validated for initial use.

The documentation requirements need to be in accordance with the ISO 13486:2016 standards. The organization is responsible for documenting the QMS to ensure effective operation and control of the implemented processes. The documentation also must include the scope of the QMS, and a description of how the processes interact with it. Documentation also includes keeping track of medical devices. The organization needs to ensure they establish files that include general description of the medical device, intended usage, labeling, and specifications for the product, installation, monitoring, handling, distribution, and servicing. Furthermore, it is up to the organization to establish methods for protecting confidential health information; Plus, the standard requires that records remain legible, identifiable, and retrievable.

ISO 13485:2016 also defines several terms, most notably an implantable medical device (IMD), and a medical device. An IMD can only be removed via medical or surgical intervention. The IMD is intended to be completely or partially introduced into the human body. Additionally, the IMD is also intended to replace an epithelial surface or the surface of the eye and remain after the procedure for at least 30 days. Meanwhile, this standard defines a medical device as an instrument intended to be used alone or in combination for one or more of the medical purposes of the following, which include but are not limited to:

- diagnosing, preventing, monitoring, treating or alleviating a disease or injury
- supporting or sustaining life
- control of conception
- investigating replacing, modifying or supporting anatomy of a physiological process

The ISO 13485:2016 standard is an internationally recognized gold standard for the medical device industry. The gold standard shows potential patients and clients that quality is highly valued, and that the medical institutions have a strong framework established to ensure high quality is continuously achieved. This would enable potential patients to be more confident they are getting the best care possible, leading to more trust in medical IoT and ultimately a better quality of life.

#### 6) ISO/IEEE 11073 PHD

IEEE 11073 PHD declares how personal health devices should communicate with each other to exchange healthcare information. The standard also discusses how each entity should behave and presents a set of device specializations with properties of each medical device. In short, IEEE 11073 PHD defines procedures and messages for data exchange between a device and an external system for each personal health device. This involves several technologies, mainly a personal area network (PAN), Bluetooth, and Zigbee. Since the standard uses a PAN, the standard can achieve lighter and cheaper communication. This is perfect for personal health devices since they have small computing power. Unfortunately, the small computing power comes with a cost: the amount of data it can hold is very small. While the IEEE 11073 PHD standard cannot contain patient information. Furthermore, if a system wants to obtain detailed about a personal health device, an additional step is required. The standard does not provide a standardized output format that can be used by applications wanting to access data via software interface. These drawbacks limit the standard’s applicability in IoT environments, which may not be applicable for medical IoT because patient data is crucial to ensure the patient gets the best quality healthcare. Since it cannot contain patient information, extra work would be required, leading to extra resources having to be expended.

### C. CHALLENGES AND FUTURE PROSPECTS

Seven medical IoT architectures were reviewed. First, the edge computing architecture is beneficial due to low cost, inclusion of 5G, WBANS, and home monitoring ability. It is important to consider the ability of the architecture to reduce system-wide costs in future research. The second architecture discussed is the MeDIC architecture which is ideal for reducing network traffic and response time and should be considered in future IoT research. Then, a blockchain-based smart contract is discussed with the purpose of ensuring security and effective data management. Limitations of smart contracts that should be addressed are consistency, immutability, transaction processing speed, and scalability.

Fourth, a security-oriented design framework is analyzed focusing on security from a hardware standpoint. The proposed system is highly adaptable as reusability is accomplished, but the architecture was not evaluated against different types of hardware attacks, questioning the true adaptability of the architecture, and creating a new avenue for future research. Next, a Bluetooth-based architecture was discussed involving the COVID-19 pandemic and contact-tracing, while maintaining privacy. The architecture was effective, possibly because of the restricted scenario due to the pandemic. Sixth, an energy efficient fog-to-cloud architecture was discussed, which uses a combination of online and offline access, quick real-time response analysis, fog node or IoT gateway, and reduced energy consumption. Finally, a LoRa-based architecture was analyzed, and the high power consumption due to Wi-Fi, partial mobility response, limited range of data receiving/transmitting, and interferences pose topics to be addressed with continued experimentation.

It is important to understand relevant standards for medical IoT. IEEE 802.15.6 is important for diagnosing and regulating patients using exceptional service and low power consumption. IEEE 802.15.4 has recently improved, but still needs to expand communication over large areas. Recently, there is becoming an increasing need for interoperability, which is a challenge for the FHIR standard. Another challenge of FHIR is backwards compatibility, but with continued improvements, these challenges will inevitably be remedied. FHIR also uses simple API’s, eliminating the challenge of complexity, but for OpenEHR, the complexity is greater, demanding new methods for resolution. The ISO 13485:2016 seems to be the model standard in the healthcare industry due to its many benefits. Finally, the ISO/IEEE 11073 PHD standard is perfect for personal health devices, but the amount of data it holds is minimal.

## VI. APPLICATIONS OF BIOSENSORS FOR MEDICAL IoT

### A. FOG COMPUTING FOR THE ELECTROCARDIOGRAM (ECG)

**FIGURE 9.**
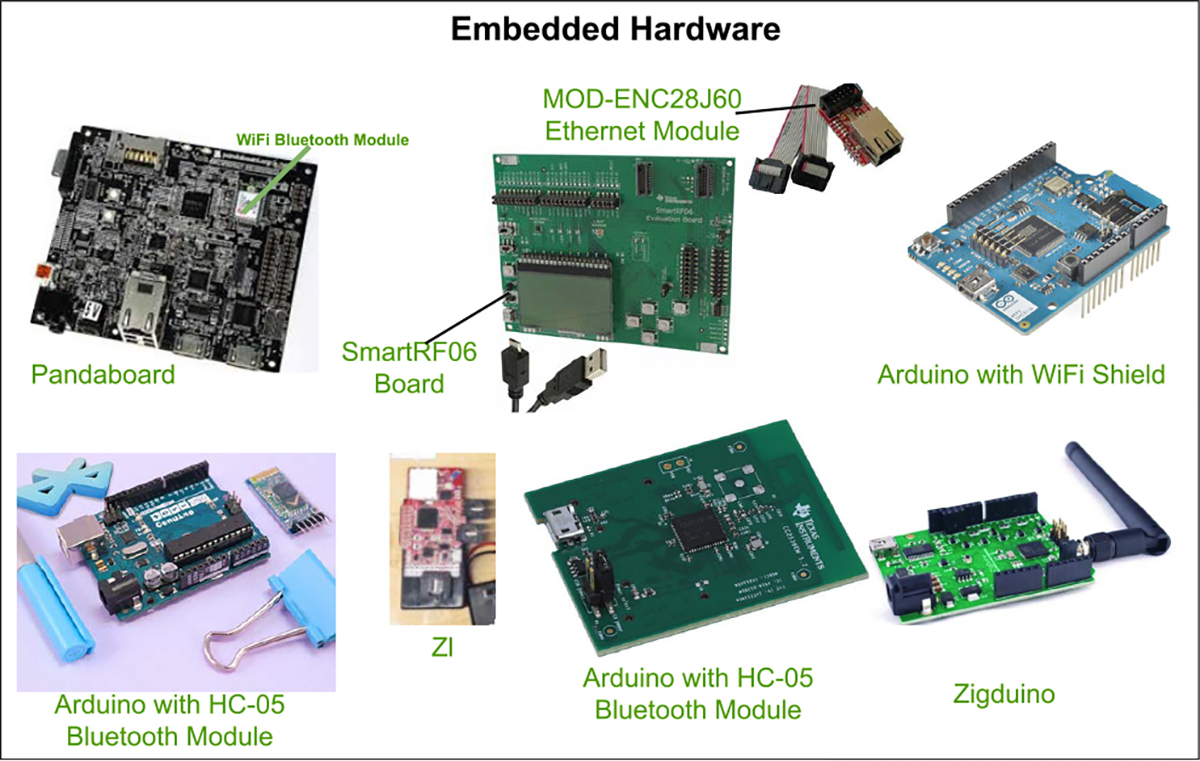
Embedded hardware of a proposed IoT based fog healthcare gateway [166].

The following article [166] is a case study using fog computing for the Electrocardiogram (ECG).The case study works to transition the following traditionally cloud-based services to a more fog-based system. The system consists of mining data, handling storage, performing notification services, detecting surroundings, and a GUI. The findings revealed that fog computing can help produce a 93% data size reduction resulting in a more efficient utilization of network bandwidth and can lower latency by 3.5% to 48.5%, offering a more real-time response. Combining various embedded hardware used in this case study are seen in Figure 9.

### B. ALZHEIMER’s DISEASE

Here, the authors argue that medical IoT can also be used for monitoring symptoms related to Alzheimer’s, such as lower judgement, loss of memory that can disrupt the patient’s regular routine, etc. [167]. Using medical IoT would benefit not only those with Alzheimer’s but the caregivers as well [167], [168] proposed applying chips to be embedded in the patients’ clothes (inconspicuously) to track of their position. Other sensors that are used could keep an eye on patients’ whereabouts plus their travel patterns, and medical professionals can grow aware of dangerous scenarios [167]. Sensors can possibly be positioned in areas to detecting patients’ movements and health signs. If the patient grows more disoriented, sensor technology could notice that pattern. If the patient becomes agitated or irritable, the sensor can be applied towards detecting elevated heartrates and other vital signs. Table 5 represents components needed in the author’s framework for applying IoT to Alzheimer’s Disease detection [168].

### C. FALL DETECTION

**FIGURE 10.**
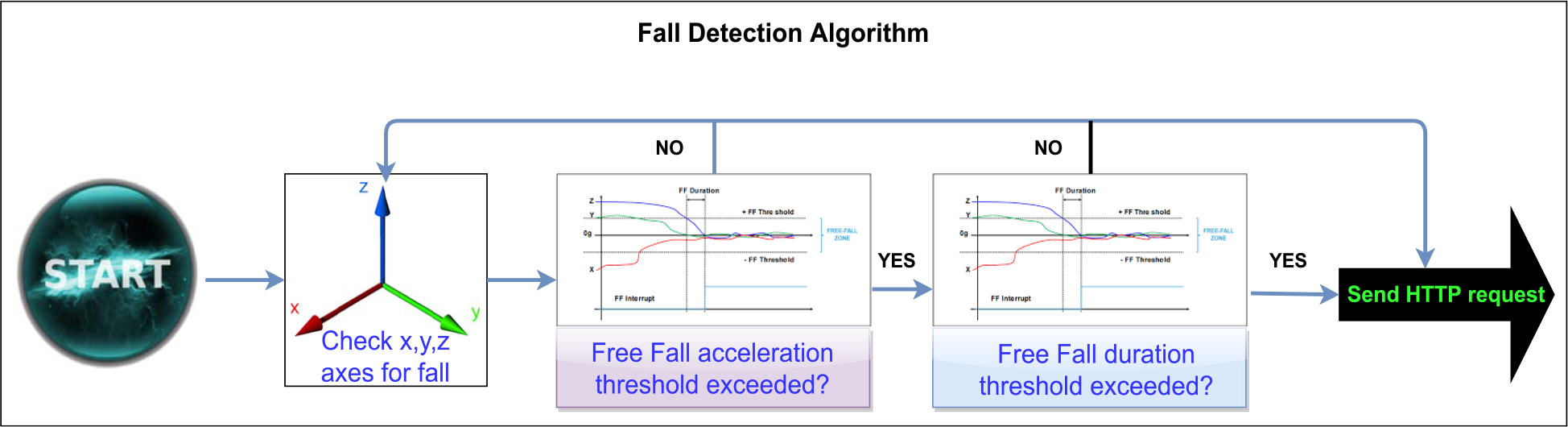
Algorithm of fall detection for IoT [169].

Another use case of medical IoT has been seen in Fall detection. The authors of [169] proposed a fall-detection system aimed toward older adults. The system was designed with a main control center for communication which is responsible for processing information, making decisions, and communicating any messages. The main control center will act as a guide between all elements of the architecture. It is composed of a server, a device for detecting falls, an Amazon Echo device, speakers, and a video camera. The authors chose the Amazon Echo because it can recognize voices and determine the best course(s) of action depending on given input. With the Amazon Echo, there is a level of convenience for the user that allows them to confirm if a fall had occurred. The architecture uses a hardware model for the system’s central server. The method for detecting falls in a house also uses an algorithm which is shown in Figure 10. The algorithm works via inputs based on a threshold. Two thresholds are used: one for acceleration and another for fall duration. When an acceleration greater than the limit established is met with a longer duration than the free fall time period, an interrupt is produced. The interrupt communicates a message to the main server of the IoT-based framework [169].

Unfortunately, some things to consider when designing IoT applications for detecting falls is the trade-off between sensitivity and specificity. Specificity determines the ratio of negative scenarios that were accurately predicted, while sensitivity determines ratio of positive scenarios that accurately happened. An ideal system would contain a high amount of both. While the authors acknowledge their implementation may not be the most efficient, it can be used as a groundwork for implementing fall detection applications for the medical IoT in the future, and their work was able to reduce the rate of false positive [169], [170].

### D. SMART HOSPITALS

Smart Hospitals are a new topic in medical IoT. Statistically speaking, researchers forecast that by 2024 the smart hospitals market-share can be worth $ 63 billion [171]. Smart Hospitals will offer the following benefits: patient engagements, streamlined communication, optimized workflow, data analytics, and asset tracking.

Smart hospitals are crucial for both medical IoT and Healthcare-IoT because systems need to be efficient to reduce patient casualties [172]. Usage of IoT in hospitals is a recent trend that can facilitate better and more personalized treatment, with a better quality of life for the patients [173]–[178]. In fact, several works were able to use smart hospitals for monitoring more serious diseases such as epilepsy [179], [180]. Despite all the promise of smart hospitals, there has yet to be an architecture for them, or even a way of evaluating the performance of a smart hospital [181]. These elements are crucial to implement for smart hospitals in order to increase their marketability and guarantee more usage in the years to come [175], [182]. However, there are some places which have already begun to implement smart hospitals as seen by Table 6 [172].

Some authors have proposed a model of evaluating a smart hospital’s performance. The authors of [183] proposed using analytical models to evaluate smart hospitals [184]–[186]. Specifically, they use Stochastic Petri Nets (SPNs) [183]. These are concepts that represent complicated architectures with unique properties. SPNs have probability models which can allow us to estimate how well a system will perform. The SPN model is able to allow the configuration of 13 factors, allowing us to consider more alternative scenarios. So this model can come in handy for smart hospitals so they have a solid foundation [183].

In another work, the authors proposed a smart hospital architecture using the Narrowband-IoT (NB-IoT), which is shown in Fig. 5 [187]. In doing so, they were able to perform a case-study where they use this for monitoring quantitatively how much of a drug is used during infusion. This is a relevant issue in hospitals because the medical personnel cannot monitor the infusion constantly [188]. NB-IoT is a lower-powered standard for wider areas that works practically in any if not most locations. This particular protocol has benefits of low pricing and reduced consumption of power, which can enable new devices to connect to gadgets that need to use smaller quantities of data over a certain amount of time. The authors note that while NB-IoT can provide many benefits there are drawbacks as well [187]. It has high latency and poor mobility. The reason for its poor mobility is because the NB-IoT Standard does not account for mobility or latency. Nevertheless, the authors mention the advantages of using NB-IoT such as: more amount of space, broader range, consumes low quantity of enery, and low price/tradoff.

In [187], they go over each of the functionalities presented in the their framework. Most notably, they were able to use this to design their own monitoring system [187], [188].

More recent works, such as [189], [190] have proposed using 5G for smart hospitals as well. However, high speed, low latency, spectral efficiency, and low energy consumption are the requirements of a 5G based modern hospital. Unfortunately, smart hospitals are still in infancy stages, and their requirements will likely be much different in comparison to a regular hospital. Additionally, the quality of 5G will depend heavily on network infrastructure as well, but cost is another potential drawback of incorporating 5G into smart hospitals, so one of the important goals for incorporating 5G into smart hospitals should be to minimize cost as much as possible. Furthermore, 5G alone may not be sufficientl; other technologies would need to be incorporated to ensure optimal operation is achieved [191].

### E. ARCHITECTURE OF AN INNOVATIVE SMART T-SHIRT BASED ON THE INTERNET OF MEDICAL THINGS PARADIGM

In [192], the authors present a medical IoT system based on a wearable device embedded on a T-shirt. The system addresses several challenges in medical IoT, such as developing medical IoT systems which fuse data provided by sensors, implementing compression algorithms that can reduce the amount of the data transmitted to a server, and implementing high conductivity textile materials that are washable. The authors’ proposed a smart T-shirt called S-WEAR. S-WEAR can measure vital parameters on the body, trace their position via indoor localization techniques, and posture (lying down, standing, walking). S-WEAR is also able to handle scenarios where the user is not at home. In that case, S-WEAR measures vital parameters of the person, and it traces the position through the Global Navigation Satellite System (GNSS).

The authors discuss the architecture of S-WEAR. In general, the framework for S-WEAR consists of five modules: the smart T-shirt, which embeds all the electrodes used for acquiring the ECG signals, the bio-impedance (Bio-Z) measurements and the two skin temperatures, the core module (C), the extended ECG module (E), the position measurement module (P), and (v) the Internet interface module (I).

S-WEAR also embeds sensors for the following: ECG monitoring, respiration rate measurement, (3 skin response, skin temperature measurements, and activity classification and monitoring. S-WEAR also has a microcontroller which is supposed to do the following activities: acquires the measurements provided by the sensors, stores them in a SD memory card, and sends them to an S-BOX via a BLE interface or to a Server via a Wireless Wide Area Network (WWAN) interface. The S-BOX is a station which communicates with the S-WEAR via a BLE interface and is connected to a DSS (Decision Support System) via Internet. The S-BOX needs to acquire measurements provided by the S-WEAR shirt and store them on memory for a long amount of time; Then it needs to integrate all the provide information by applying data-fusion algorithms. The S-BOX also must perform analysis to detect any potential anomalies. The DSS is a centralized system that receives information from the S-BOX and the signals related to the detected anomalies. The DSS also predicts emergency situations and transmits the information related to the emergency alerts to a monitoring station, which is connected to the Internet.

Biosensors are the starting point for medical IoT, they feed biological data to the cloud. These sensors are intimately connected to the human body either through direct contact with the skin (non-invasive) or located within the human body (invasive). Throughout this paper we spoke extensively on using biosensors in the architecture of medical IoT, but never elaborated on these biosensors. Here we will tackle the applications of the vast array of biomedical sensors from a hardware and medical perspective.

### F. GLUCOSE MONITORING

Glucose monitoring for diabetic patients was naturally one of the first applications of implanted biomedical devices. These devices have successfully aided in providing quality care to diabetic patients. They have evolved from simple hardware with a screen, to M2M devices, to IoT IPv6 linked devices where data is accessible by family and doctors. Diabetes is a condition that occurs when one’s blood sugar is too high. Continuous monitoring aids in this treatment regimen by providing more accurate results in real time. While the devices themselves may not be more accurate in any instance, the averaging their continuous stream of results is. The CDC reports 9.8% of the adult population has type 2 diabetes, and 1 in 4 adults did not know they had the condition [193].

Type 1 diabetes is juvenile-onset diabetes, and often requires continuous monitoring of glucose levels. These monitors are invasive and require some level of epidermal (skin) penetration. Type 2 diabetes is adult onset diabetes, and is often taken less serious and not monitored depending on the severity. Gestational diabetes is high blood glucose levels during pregnancy. Those with type 2 diabetic or gestational diabetes would benefit from continuous glucose monitoring, but the invasiveness of continuous monitoring is a deterrent. Patients with type 2 diabetic or gestational diabetes will often opt for occasional blood testing instead.

This highlights a recurring theme in the limitations of medical IoT: hardware limitations. The ideal glucose monitoring biosensor is stable, non-invasive, accurate, and does not have the necessity of calibration for the device [27]. Non-invasive glucose sensing in real-time are in the works, and we have reason to trust its success given the lucrative prospects. A practical non-invasive glucose monitoring would be beneficial to anyone and could help with early detection of type 2 diabetes. The potential of a non-invasive glucose monitor is explored extensively in [194]. The hope is that these non-invasive glucose monitors will become as commonplace as non-invasive wearable IoT heart monitors.

### G. ELECTROCARDIOGRAM MONITORING

There is an array of simple heart monitoring devices on the market, but the monitoring of the electrocardiogram (ECG) is more finely defined as the monitoring electrical activity usually recorded by an electrocardiography. This is usually administered non-invasively during a physician visit over the course of a few minutes. Electrocardiograms detect abnormal heart rhythm, but a major limitation is that they can only detect abnormal heart rhythm during the test. An abnormal heart rhythm could occur before or after the test and the physician would not know. Wearable electrocardiogram IoT recently entered the market [195], and is a workaround to this limitation. The continuous and real-time data from the hearts electrical activity is securely stored in the cloud and can be sent as a report to the patient’s physician. This data could also be continuously monitored for abnormalities [195].

### H. TEMPERATURE MONITORING

The heating up of our bodies core temperature is a defense mechanism for fighting off the viral infections as these infections have trouble surviving in higher temperatures. Early detection of viral infections is possible through continuous temperature monitoring. Noninvasive body temperature devices already exist and are commercially available. Use of continuous body temperature monitoring in commercial medical IoT has been limited to infants, as this age group is more susceptible to death by viral infection. Monitoring only body temperature alone in adults is lackluster but combining this with the monitoring of other vital signs could prove more useful.

### I. ELDERLY MONITORING

Many elderly individuals have various impairments that could benefit from IoT monitoring. In the past there have been significant human resources dedicated to caring for the elderly. This has become a particular concern in countries with an aging population, which often congruently have a shortage of age-related specialists. Some of this burden on caretakers could be alleviate by a smart IoT system. The feasibility of a smart IoT system for the elderly has been shown to achieve promising results in preliminary experiments and clinical trials [185].

Falls can be considered the most prominent cause of deadly injury and also the most typical reason for non-trauma-related hospital treatments among older people [196]. Detecting falls is another promising medical IoT that could benefit the elderly and others. This technology is becoming standard in wearable smart technology [197]. If a fall is detected the user’s smart devices asks them if they are okay or if emergency services need to be contacted. If the user remains motionless for a set period, the devise will automatically contacts emergency services throughout the cellular network. Falls are also recorded and historical data will likely be viewable by a physical in the future which could aid in making of medical decision. It is noteworthy that this fall detection IoT could benefit anyone and will likely be adopted by all as they become more normalized. In the future this technology could adapt to contact emergency services immediately when a car accident or other trauma is detected.

### J. MEDICATION MONITORING

One of the most clear-cut areas of medicine where an IoT infrastructure could benefit is the distribution and dosing of medication. Overdosing and under dosing, i.e., medication noncompliance, is a common and costly issue that is already being tackled by simple reminder focused smartphone applications. However, the future medical IoT may go beyond simple reminders and have more intimate link to some disruption and dosing hardware. Medicine boxes linked to a smart IoT network would know with certainty if the patient took their medication [198]. Often hardware is the limiting factor in medical IoT, but here two juvenile hardware technologies are needed: (1) a scale in each capsule to detect if medication is present, and (2) some locking mechanism in each capsule to prevent the patient from taking the wrong medication.

The opioid epidemic is a recent exponential growth the deaths caused by opioids. In the United States the opioid epidemic claimed the lives of 47,600 in 2017 compared to 8,048 in 1999 [199]. This epidemic is caused expectancy in the United States to dropped again. Though many of these deaths are from illegal opioids, the problem often begins from an opioid prescription or illegal prescription sharing. A smart IoT medical box could counter this epidemic by: (1) capsules would have a regulating daily capsule output, preventing patients from overdosing on opioids, and (2) unused capsules would not be accessible, preventing them from going on the black market. A government-controlled system may require these medical boxes to be returned. Some failsafe would have to be implemented, as a service outage or error could result in a life-or-death situation if medication is inaccessible.

In [200] an intelligent pharmaceutical packaging with communication capability is proposed, and it is enabled by the following components: (1) a passive radio-frequency identification (RFID), (2) a unit that uses Wi-Fi, (3) a receiver, (4) the high-resolution weight bridge sensor, and (5) a tablet with extension ports. This intelligent pharmaceutical packaging integrates with other wearable medical sensors proposed in [200].

### K. CANCER MONITORING

Cancer’s another cause of fatality in United States. Early detection is still one of the more effective counters to many forms of cancer. Since the 2000s wireless biomedical sensors have been proposed for early detection of cancer cells. There is ongoing research into detecting changes in protein levels that could be an early sign of cancer detection [201].

Field-effect transistors made from the material Silocon (SiNW-FETs) are a technology showing much potential in cancer detection. One of these uses a modified electrode to detect cyclin A2 [202]. Electrochemical sensors can find certain elements in cancer cells and distinguish between cells with cancer and cells without cancer. Additionally, these sensors can monitor the effectiveness of anticancer medication by monitoring the protein levels. Others have found that cancerous cells excrete certain compounds the effect blood flow [203]. By monitoring the blood flow in suspicious areas, physicians could be given an earlier warning window of precancerous conditions.

Those with a family history of certain cancers may benefit most from some form of targeted invasive monitoring. As the price of genetic testing decreases, a future where patients know what cancers they are genetically predisposed may be commonplace. Invasive biomedical IoT would target the specific secretions of the cancer determined to be at high risk to the patient given their medical history, genetic history, and age [204].

While medical IoT’s benefit is attributed to the communicating pertinent medical information with the outside world, there are more locally flavored implants that benefit more from communication with each other [205].

### L. ARTIFICIAL RETINA

Over the past two decades many have taken on ambitious projects in chronically implanted artificial retinas to help those with severe visual impairments reach a minimum level of vision [206]. These artificial retinas often involve an M2M network of hundreds of micro-sensors in constant communication with each other. The hardware of artificial retinas can be divided into two distinct components: (1) an integrated circuit and (2) a network of sensors. Early keystone work on artificial retinas dates back to 2000.

In [206] an integrated circuit of 100 micro-sensors layer out in a 10 × 10 grid was used. These micro-sensors has both transmit and receive capabilities. They were coated in a biologically inert substance. When exposed to a light stimulus the sensors promote a normal chemical response that mimic that of a heathy retina.

The Argusa II is the only approved artificial retina for marketing in both the United States and Europe. This artificial retina takes a different hardware approach. The Argusâ II uses a camera mounted on tinted glasses to send visual data to an electrical device implanted behind the eye. Nine of thirty initial participants had serious side effects including low intraocular pressure, inflammation within the eye, and retinal detachment.

Implanted biomedical sensors in a multimode system have certain advantages over other networks. The fixed placement within or on the body can be exploited, as there is no need for the burdensome proximity monitoring or neighboring node monitoring.

### M. TUBERCULOSIS

Tuberculosis is a highly contagious disease caused by bacteria spreading via air from across people when they coughs or speaks [207]. If not taken care of accordingly, it can be deadly. This disease often occurs in the lungs. The growth rate of the disease is said to grow by 0.4% but is most prevalent in South Africa. Slow detection of it is because of slow methods for finding tuberculosis and even longer duration of treatments and therapy [29]. As a result, the patients can develop a case of drug-resistant tuberculosis, which is more difficult to combat. In these scenarios, the application of biosensors can come in handy. Biosensors allow molecular typing to reduce further incidents. One example [208] has managed to develop such a biosensor, and it was able to not only detect Tuberculosis but also genetic mutations.

### N. POROUS BONE

Also known as Osteoporosis, It is an illness where bones become more fragile, resulting in weaker cartilage among individuals [209]. Usually, density of one’s bones is quantified via radiology techniques, but these have several disadvantages such as the radiation, cost, and the requirement for staff to be knowledgeable of such methods. Therefore, biosensors would be excellent candidates to remedy this because they can potentially be less costly and the technology for them is more advanced. Recently, a biosensor was successfully developed to catch early diagnosis of osteoporosis and monitor related drug treatment [210].

### O. BACTERIAL INFECTION

This type of bacterial infection is called Urinary Tract Infection (UTI), which is among the most common bacterial infection. Statistically, it accounts for over 30% of infections noted by hospitals [211], [212]. Almost 1/2 of individuals will probably deal with one in their future; Patients suffering from recurring, difficult UTIs may experience three episodes of infection or more per year. An average diagnosis of UTI typically takes between 2–3 days, which has the potential to be lessened due to biosensors [211]. There are in fact biosensors being developed to detect UTI much earlier. UTI is rarely fatal, but it still affects all patient demographics [213]. The new generation of medical devices offer the chance to diagnose UTI much quicker without using a lot of power. One use case of bio-sensing technology applied towards a bacterial infection is known as a Sensing Probe. This device utilizes many bacterial probes as recognition components. The Sensing Probe has sixteen sensors to permit versatility and lower background noise. In tests with the affected person the device had 92% general awareness as well as 97 % specificity [211], [212].

### P. COVID-19

Recently, there has been an influx of research regarding COVID-19, its inner mechanisms, and prevention [214]. Remote healthcare and monitoring will now be more important than ever to ensure patients’ wellbeing. The work of [215] discusses how biosensors could be used for COVID-19. According to [215], for biosensors to be useful in pandemics, they need to have certain characteristics. These characteristics include being cost effective, easy to use, having a long shelf life, disposable, having a quick response time, high specificity, selectivity, etc. Additionally, several types of biosensors could be more suitable for detecting COVID-19 than others. These types of sensors include electronic sensors and optical biosensors.

Applying biosensors for COVID-19 is a highly promising area of research and development, and there have already been several studies attempting to accomplish this. In [216], the article discusses PoC (Point-of-Care) biosensors for COVID-19. PoC biosensors are popular due to their low cost and user-friendliness, allowing for faster medical diagnosis. Some examples of PoC biosensors include chip-based and paper-based biosensors, both of which had advancements, and have been used for rapid diagnosis of infectious diseases. However, paper-based PoC biosensors have attracted more attention due to their cost-effectiveness, biodegradability as well as ease-of-fabrication, functionalization and modification. Additionally, [217] discuses the possibilities of using optical biosensors for COVID-19 detection and [218] discusses the implications of using nano-carbon based biosensors for COVID-19 diagnosis. It is expected that development of biosensors for COVID-19 will have to focus on cost-effectiveness and speed in order to achieve fast and reliable diagnosis.

### Q. CHALLENGES AND FUTURE PROSPECTS

The idea of Fog Computing can effectively utilize network bandwidth and lower latency offering real-time responses. Alzheimer’s Disease can be detected using sensors to monitor patients’ movements, identifying disorientation. Another use case in medical IoT is a fall detection system that produces an interrupt if an acceleration threshold is exceeded, indicating a fall. There is, however, a trade-off between sensitivity and specificity, decreasing the reliability of the system. Future work can determine new methods to increase the reliability of determining patient fall instances.

When monitoring serious diseases, the implementation of smart hospitals could be a potential solution. Smart hospitals are not yet a common practice, but future research can increase the accuracy of their practices. For example, the Narrowband-IoT structure has benefits of low power, low pricing, low energy, etc. The challenges opposing these benefits are poor mobility and high latency. Future research may address the latency issue, which is critical in healthcare settings. Finally, the S-wear smart t-shirt was discussed and it’s ability to monitor ECG data, respiration rate, and activity. The shirt, in theory, is an ideal way to non-invasively monitor important health aspects, and future research could improve the detection ability of the sensors.

The application of biosensors is a crucial topic in medical IoT, and eleven of those applications are discussed in this article. First, current applications of glucose monitoring, especially continuous glucose monitoring, is invasive. In the future, a non-invasive monitor can potentially detect diabetes early-on for better management. ECG sensors are common and produce continuous, real-time data that is useful in detecting heart abnormalities. Non-invasive body temperature sensors could be useful in the infant population since a higher change of mortality exists. In the elderly, perfecting fall-detection systems could prove to be a beneficial choice of research, minimizing serious injuries. Medication monitoring devices could potentially reduce risks of overdose, especially in children, and increase adherence. Precancerous conditions can potentially be detected early by using sensors to identify certain compounds excreted into the blood.

Artificial retina’s using sensors have been constructed. While only the Argusa II is approved for use, improvement is necessary because 9 out of 30 participants had unwanted side effects. Biosensors have been proven effective to detect tuberculosis as well as genetic mutations. Early osteoporosis and related drug monitoring have been proven to be effective using biosensors. Using a sensing probe, UTI’s can be detected. Electronic and optical biosensors are possible choices for the detection of COVID-19. Since the emergence of COVID-19 is recent, more research can be conducted to find the best biosensors for optimal detection.

## VII. CHALLENGES OF MEDICAL IoT

Implanted medical IoT refers to the various kinds of implantable medical devices that are part of medical IoT. Implanted medical devices have been actively used for treatment and diagnosis of various diseases [219]. These implanted medical devices can include but are not limited to pacemakers, insulin pumps, hip implants, implantable cardiac defibrillators (ICDs), implantable biosensors, and others. For the purposes of this section, we will be focusing on implantable biosensors.

Implantable biosensors have been quite popular due to their ability to continuously monitor patients with minimal patient intervention. Implantable biosensors also have promising potential for usage in diagnosis, monitoring, management and treatment of various diseases [63]. Like biosensors, implantable biosensors have criteria that needs to be met so that they can be used effectively and ensure the patient does not experience any complications from these implantable biosensors [220]. These criteria include:

- Specificity & Sensitivity - The implantable biosensor should operate within acceptable therapeutic range while in the presence of complex solutions.
- Biostability - Here, it is imperative that the patient does not experience any negative discomfort with the device. Any negative immune reactions could cause the device to become non-functional.
- Transmission - Signal output(s) that do get transmitted need to be in such a way that can help both the patient and clinician.

However, sensors used within the human body face unique design challenges compared to the sensors used in traditional IoT [221]. Indeed, designing an implantable biosensor that is safe despite tissue being exposed to: (1) continuous heat diffusion, (2) continuous wireless transmission, and (3) a foreign material is still a challenge. On top of this, any hardware malfunction, or need to replace a power supply, will result in an undesirable and costly surgery [222]. Long term damage to the surrounding tissue is possible if proper oversight is not given. The effects of any new prolonged implantation may requires months or years of trials before public use. Compare these to the procedure for replacing or releasing traditionally IoT, and one notes the laxer nature [223].

### A. INVASIVENESS

The technology for invasive monitoring of vital signs, proteins, and other chemical levels is commonplace. However, invasive medical IoT has a new set of challenges that have prevented them from being commonplace: (1) penetration increases the risk of infection, (2) a medical professional may be required for installation, and (3) the idea of an invasive device is offsetting. These devices will likely be connected to the patients cellphone with Bluetooth for continuous connection to the internet as opposed to connecting the device directly to some WiFi [224].

Additionally, medically implanted sensors should be as robust and fault tolerant is possible as it is undesirable to surgically adjust these sensors. One faulty node should not cause the distributed network to faultier. Other nodes should remain functional despite the fault of one node. Each node should have some level of independence, while still having cooperation capabilities with other nodes when necessary. The power consumption should also be reduced by appropriately reacting to the death of a node [63].

### B. POWER

Low power is also a concern for both medical IoT and other applications of commercial non-medical IoT. This lower power concern is not only a result of the small size of the implantable biosensors and medically implanted devices, but also a result of the lack of a constant power supply. Batteries are one possible solution, but once battery power runs out the medical IoT system seizes to be a network. To prolong battery life, one is limited to either: (1) inventing a better battery, (2) implanting a bigger battery, or (3) lowering the power consumption of the device. The battery power options from advancements in commercial non-medical IoT will carry over to medical IoT. Where these technologies diverge is when medical IoT is implanted. It is impractical to remove the implant every time a new battery is needed. Additionally, the bodies potential incapsulating reacting to the biosensor may make it difficult to simply replace them. While wireless charging of these implanted biosensors is certainly possible, the heat concerns from this are technology are still a deterrent [225]. It may be beneficial to implant multiple nodes within a patient forming a medical IoT network. Each node will have responsibility such as sensory processing, information processing, and communication. It is ideal for each of these nodes to consume power evenly, so that simultaneous recharge is possible [225].

### C. HEAT

It is known that regional prolonged heat exposure, even on superficial areas, on the body can cause damage surrounding tissue. This is of particular concern for implantable biosensors, as some level of dissipated heat is inevitable. The tolerance of heat dissipation depends on the amount of heat dissipated, the area of the implant, and the duration of the implant. There is a great amount of uncertainty what the effects of low heat over the long term will be. One area of concern is heats effects on enzymatic reactions [226]. Some biomaterials, such as glass, are seemingly inert and safe for internal use, but other properties, like poor thermal dispensation, make them unusable for heat prone biosensors. This causes the glass to store the heat concentrating all the heat dispensation in the sensor. The presents two problems: (1) the sensor could overheat, and (2) the head is concentrated on certain tissue.

### D. MATERIAL

Design restraints such shape, size, heat dissipation, and materials of implantable biosensors will be more restrictive. For implantable biosensors, hardware is usually the limiting factor, because the hardware has to cope with various computational requirements while still functioning safely in the body. Even the most robustly designed software and hardware is of no use if it cannot function safely and effectively within the body. This adds a whole new layer of not only hardware limitations, but legal limitations.

The human body’s immune system is not naturally receptive to the insertion of foreign entities into our bodies, and will actively combat the intrusion. Biocompatible materials are known. However, if the body considered the medial IoT a threat there is a risk of it becoming incapsulated in fibrous tissue. Another threat is thrombosis, where the red blood cells attach to the surface of the sensor never time. Toxic or hazardous substances could result just from the bodies interaction with the implanted biosensor [63].

The concerns highlight the importance of picking biocompatible materials. Common biocompatible materials include synthetic polymers, biological polymers, metals, and ceramics. Not all biocompatible materials will be suitable for the intended biosensor, so the needs of the biosensor must be taken into consideration when picking a suitable material. Other materials such as glass were noted as being seemingly safe biocompatible material, but their poor heat dispensation causes other issues making it unsuitable [220].

Other material concerns have expected natural growths that occur over time that may be beneficial. Silicon substrates can form a layer of thermally conductive diamond or aluminum nitrate [227]. Despite the pragmatic sound of oxidation immune diamond incrusted biosensors, diamond is reactive with the iron found in blood making it unsuitable. Aluminum nitride is a far more suitable candidate.

Silicon - based material hold a lot of potential towards implanted biosensors, due to the ability of theirs to identify proteins, cancer biomarkers, small molecules, DNA sequences, as well as viruses [228]. Furthermore, they reap the benefits of ultra-awareness, selectivity, along with real-time and label-free detection abilities. Several biocompatible materials for implanted biosensors also include other materials such as chitosan, alginate, cellulose, heparin, and silk. These have been employed to act as a barrier against inner body elements such as cells, proteins, platelets, and chemical gases [220].

### E. SECURITY

Many of these implanted medical devices are usually equipped with wireless connectivity to help with the patients’ treatment and monitor their health. Unfortunately, these implantable medical devices are prone to several security issues due to their strict computational, energy and physical limitations [229]. Medical IoT collects sensitive clinical, behavioral, and lifestyle data that require robust security for data management and access. As IoT continues to grow there will be an increased strain on securing and managing this data. Frequently, these devices are going to be hooked up to a cloud for entry from anywhere.Though the prospect of stealing medical data is not as financially lucrative as stealing financial information directly, data integrity and confidentiality are still a concern [230], [231].

Even with having security in mind, medical devices often have flaws in the wireless communications they use to pass information. Providing efficient security for medical IoT is particularly challenging, because they typically have small memory and computing power, which may limit the amount security features that can be put in them. In some cases, encryption can often be absent because it risks shortening battery life of medical IoT devices. Even though medical device manufacturers are emphasizing security more, medical device companies have remained quiet regarding the security of their hardware. While this may be intended to prevent cybercriminals from gaining insight into the medical devices’ functions, it also makes it harder to gain real insight into the strengths and weaknesses of the medical device security [232]. The works [233], [234], and [235] discusses various types of attacks medical IoT devices can be prone to, such as eavesdropping attacks, malware, denial-of-service, and others. These attacks vary in severity, but still greatly compromise security of medical IoT devices.

In addition, other issues regarding the security of medical IoT also include compatibility. The work [44] also discusses this and mentions the present solutions that are used to improve security for medical IoT. It should be noted that while the proposed solutions in [44] are effective, the solutions work under a controlled environment. Security for medical IoT should be flexible in order to prepare for any unexpected security breach [236]. Another reason why implementing security for medical IoT is difficult is due to the lack of a standardized testing methodology for IoT security. In addition, the lack of universal standards on architecture, manufacture, and protocols makes it difficult to formulate a sufficient security solution that can be used generally in IoT or IoMT environments with little to no consideration to architecture [237].

Furthermore, security issues of medical IoT also impact its marketability. Wearable products are hurriedly sold in an effort to record an ever-increasing market [238]. Unfortunately, requirements for protection of equipment is not discussed, and many products don’t possess the materials or the capability necessary for the provisions of protection. Research conducted recently on customer products within IoT illustrate that products are being offered with very little or even absolutely no protection provision. Next, current investigation suggests that in reality, sold wearable products have terrible protection provisions, so assaulting them an easy task [239]. These kinds of shortcomings can cause consumers to be skeptical of medical IoT and effect marketability of medical IoT devices [240]–[242].

### F. COMMUNICATION AND NETWORKING

A major concern with wireless communication of biosensors is power consumption. The deciding factors for the power consumed is the amount of data that needs to be transmitted, the distance of the link, and how often. Zigbee and Bluetooth have shown similar energy consumption in some applications. However, in different applications there can be an order. of magnitude difference in power consumption. Therefore, designers need to consider what type of network they require, along with how much data they need to send, and what type of power source they have available and choose the protocol accordingly [243].

Additionally, one also must consider how biosensors will communicate both simultaneously and efficiently. Simultaneous communication among different types of biosensors raises concerns of congestion on nodes or transmission links. Consequently, the probability of a number of scenarios increases, such as high delay, packet losses, retransmissions, bandwidth exhaustion and insufficient buffer space.

### G. OTHER CHALLENGES

In addition to the challenges discussed here, there are still other challenges that hinder biosensors from being fully applicable in medical IoT. Because so many biosensors have been developed, a strong foundation for categorizing these biosensors is needed. This is so both the doctors and patients become familiar with the type(s) of biosensors used, how they work, and differences between other biosensors. Biosensors also must be robust. Robustness may be a more difficult challenge to address because currently, there is a lack of multi-parameter systems regarding monitoring physiological measurements. Usually, only a few parameters are addressed, but for biosensors, parameters need to be designed in such a way that is applicable, flexible, and adaptable.

Other challenges that also need to be addressed include ethical and legal challenges of biosensors regarding the patient. These challenges revolve around issues of autonomy and informed consent for the patient. Biosensors may involve therapies with aspects that are unfamiliar to patients, so voluntary and informed consent is crucial. This also extends to the user-agreements regarding data collection, which many patients have trouble understanding, and most users routinely ignore them. Frequent modifications to biosensors also highlight additional challenges - patients may not understand what changes the modifications implement. Many papers that discuss challenges of biosensors discuss technical challenges in great detail, but challenges of biosensors from a patient perspective are usually not discussed or talked about in little detail. So there needs to be more work in educating the patient about how biosensors will be incorporated into their healthcare regimen.

## VIII. CONCLUSION AND FUTURE WORKS

Recent advancements in both the field of [244] biomedical engineering and computer science have opened the door to commercialized medical IoT more intimately connected with the human body, but still many residual challenges unique to the field remain. This paper surveys aspects of healthcare, biomedical, and bioinformatic IoT technologies: (1) emergent advancements in biomedical sensors, (2) distinctions between invasive and non-invasive biomedical sensors, (3) network architecture of medical IoT, (4) security concerns of medical IoT, and (5) hardware limitations of biomedical sensors in and on the human body. We reviewed the state-of-the-art and emergent technology each aspect of medical IoT. It is not expected that researchers tackle the broader challenges listed, but this work is focused on generating intrigue for researchers to tackle specific problems in their respective fields. There is an urgent advancement in specific domains, such as hardware, architecture, and security. For example, developing a safe, non-invasive, and continuously monitoring glucose sensor would result in a plethora of new research in analytics of this data, but without the hardware we can only speculate. The author expects this survey to give a broad overview of the field to researchers, and a newfound ambition to tackle one of these limitations.

Something to consider for future directions is how to involve the patient more in medical IoT. As it stands, there has been plenty of research regarding the technical aspects and challenges of medical IoT, but there has not been sufficient effort on how to ensure the patients completely understand how to incorporate medical IoT into their regimen. Considering this aspect, the standards both discussed in this article, and other standards do not sufficiently consider accountability; Suppose a patient’s medical IoT device malfunctions; Who would be held accountable: The patient, the healthcare provider who prescribed the medical IoT device, or those who built the medical IoT device? This highlights another issue in medical IoT that has not been given much attention: usability engineering. Usability engineering is discussed in standards relating to medical devices, but descriptions are often vague, leading to difficulty on how to interpret them. For example, the IEC 62366 standard covers usability engineering to medical devices and specifies usability requirements for the development of these medical devices. Yet, even with amending the standard, no fundamental changes to the usability engineering process have been made. As such, future work in medical IoT should consider how usability-engineering play a role in designing and implementing medical IoT devices, and how usability engineering impacts medical IoT overall.

## Figures and Tables

**FIGURE 1.**
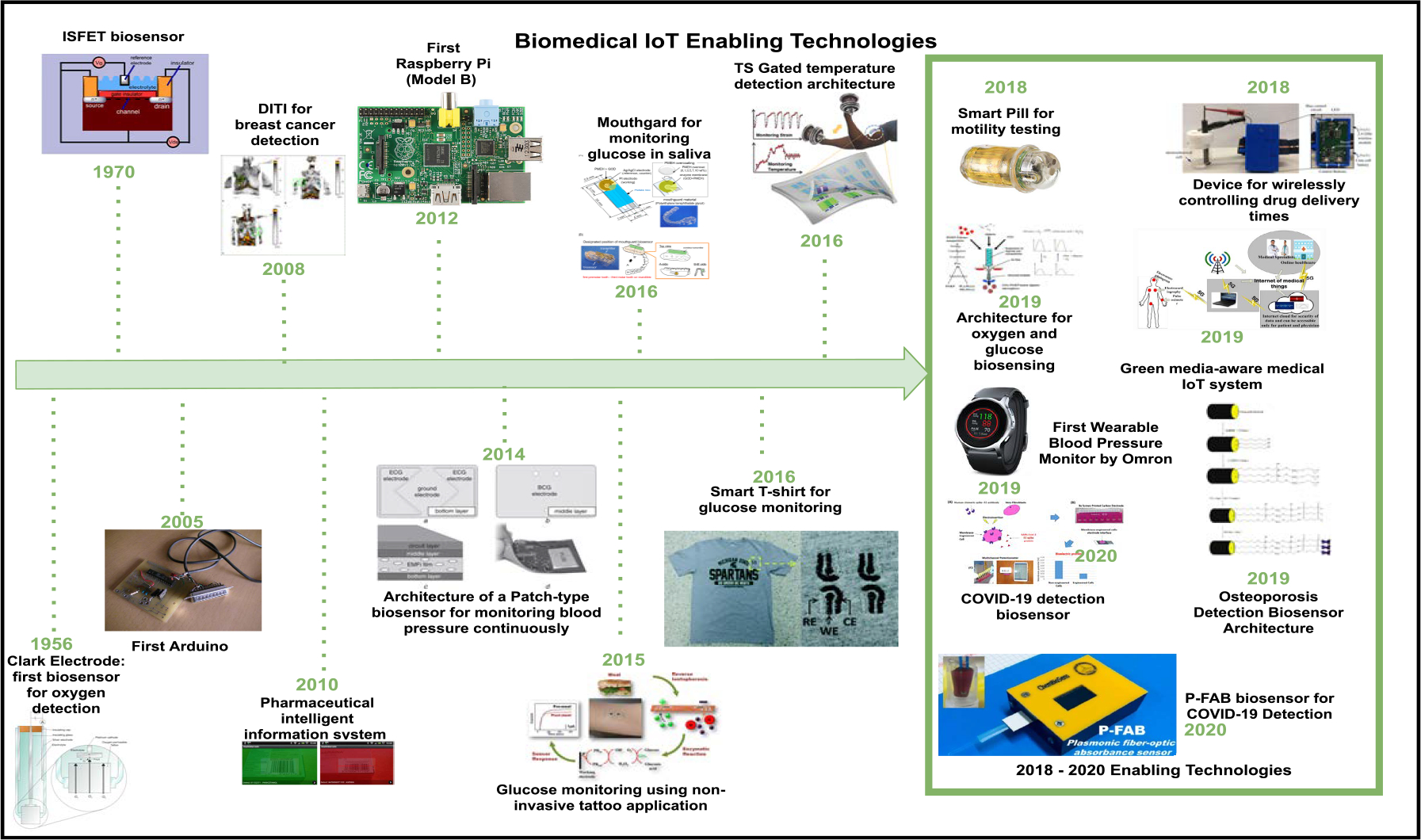
The first biosensor was produced in 1956 by Leland Clark for oxygen detection [13]. The Ion-sensitive Solid-State biosensor (ISFET) was first used for neurophysiological measurements [14]. Open-sourced hardware project Arduino released the first device in 2005 [15]. Digital infared thermal imaging system (DITI) to assist MRIs and Ultrasounds in the detection of breast cancer [16]. Three proposed information systems used to detect patient-specific medication intolerance by warning the user if a specific ingredient is detected: one barcode-based and two Near Field Communication (NFC)-based [17]. The first Raspberry Pi, Model B, a credit card-sized computer, was released in 2012 [18]. Architecture for a patch-type biosensor that attaches to the skin for continuous blood pressure monitoring using a layered ferroelectret film, electrodes, and flexible electronic circuit to measure ballistocardiogram and electrocardiogram data simultaneously [19]. Glucose monitoring using non-invasive iontophoretic-based tattoos [20]. Non-invasive glucose monitoring in the form of a mouthguard using Pt and Ag/AgCl electrodes and an integrated biosensor and wireless measurement system [21]. Electrochemical sensor woven into fabric of a smart T-shirt that uses quantitative analysis of biofluids for measurement [22]. Transparent stretchable (TS) gated Body temperature skin detection sensor for recognizing physical activity [23]. Ingestible smart pill for measuring pressure, pH, and temperature for gastrointestinal diagnostic purposes [24]. A stimuli-response drug delivery system using a wireless controller device and an electrochemical cell [25]. Green-aware medical IoT system for wearable devices [26]. Architecture for implantable devices that continuously monitor glucose and oxygen using Near Infrared Fluorophores like Pt-porphyrin to analyze specific enzymes [27]. The first wearable blood pressure monitor produced by Omron won the Red Dot award in 2019 [28]. Osteoporosis Ocn biosensor detection architecture that recognizes the presence of the osteocalcin molecule for early diagnosis using an anti-osteocalcin antibody immobilized onto a gold electrode surface [29]. Biosensor that measures using Bioelectric Recognition Assay and produces quick results for sensitively detecting SARS-CoV-2 S1 spike protein for COVID-19 diagnosis [30]. Plasmonic fiber-optic absorbance biosensor (P-FAB) used to detect the presence of COVID-19 in saliva [31].

**FIGURE 11.**
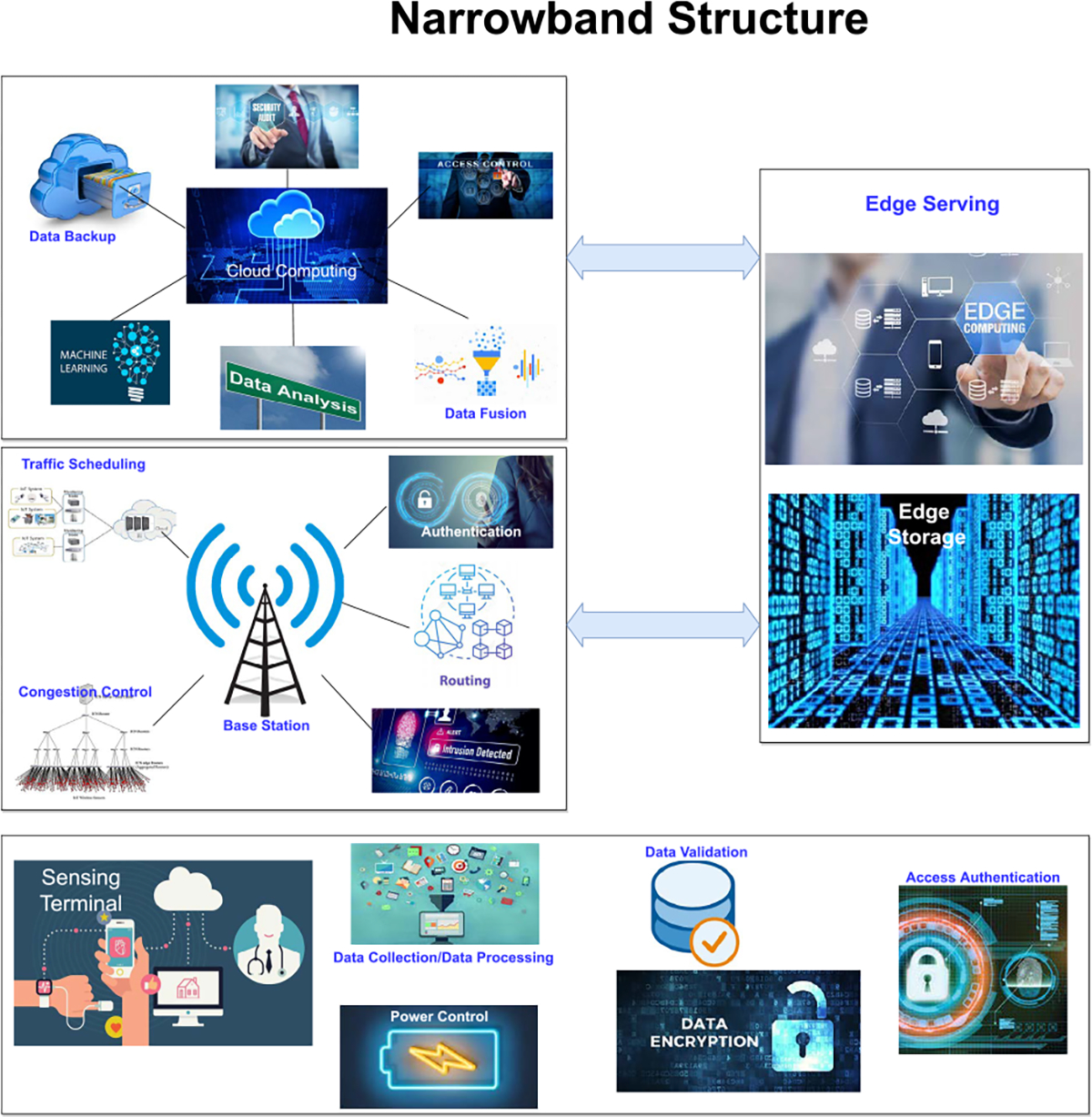
A framework of intelligent health facilities using Narrowband structure [187].

**TABLE 1.** Summary of existing surveys and reviews related to medical IoT.

Refs. (Author)	Year	Biomedical IoT (general)	Ingestible Devices	Fog Computing	COVID-19	Security	Comments
This paper	2021	✓	✓	✓	✓	✓	To give a deeper, technical understanding of biomedical IoT
Bharati et al. [35]	2021	✘	✘	✘	✓	✓	Discussed medical IoT in cloud computing
Degerli et al. [36]	2021	✘	✘	✘	✘	✓	Wearable medical devices are the main enablers of medical IoT
Albahri et al. [37]	2021	✘	✘	✓	✘	✓	Disease prevention, health promotion, and telemedicine topology mapping/architecture
Thongprasert et al. [38]	2021	✘	✘	✘	✘	✓	Discussed development of medical IoT devices for home use
Aceto et al. [39]	2020	✓	✓	✓	✘	✘	
Thilakarathne et al. [40]	2020	✓	✓	✓	✘	✓	Discusses enabling technologies and trends of healthcare IoT
Lee et al. [41]	2020	✘	✘	✘	✘	✓	Researchers examined demand preferences of medical IoT devices in vulnerable populations
Qadri et al. [42]	2020	✓	✘	✓	✘	✓	Discusses emerging H-IoT technologies and improving future quality of service of these technologies
Alshehri et al. [43]	2020	✓	✘	✓	✓	✓	Examines IoT and Al-based Smart Healthcare
Kharroub et al. [44]	2020	✘	✘	✘	✘	✓	Discussed security methods pertaining to medical IoT
Maimers et al. [45]	2020	✘	✘	✘	✘	✓	Discussed the adoption of medical IoT
Sadoughi et al. [46]	2020	✓	✘	✓	✘	✓	Discussed current medical IoT developments
Swayamsiddha et al. [47]	2020	✘	✘	✘	✓	✘	Discussed the medical application of IoT regarding COVID-19
Mutlag et al. [48]	2019	✘	✘	✓	✘	✘	Discusses healthcare applications and the use of fo
Shen et al. [49]	2019	✘	✘	✘	✘	✘	Discussed retrieval of medical IoT images
Kotronis et al. [50]	2019	✓	✘	✘	✘	✘	Discussed the evaluation of medical IoT systems from a user-perspective
McFarland et al. [51]	2019	✘	✓	✘	✘	✓	Discussed vulnerabilities, threats, and risks of medical IoT
Habibzadeh et al. [52]	2019	✘	✘	✓	✘	✓	Discussed current emerging technologies of HIoT from a clinical perspective
Mishra et al. [53]	2019	✓	✓	✘	✘	✘	Assessed existing and future technologies pertaining to medical IoT
Irfan et al. [54]	2018	✓	✘	✓	✘	✓	Discussed existing architectures of medical IoT
Gandhi et al. [55]	2018	✓	✘	✘	✘	✘	Discussed existing architectures of medical IoT and their ability to improve the quality of life
Jagadeeswari et al. [56]	2018	✘	✘	✓	✘	✓	Focused on the use of Big Data and cloud computing regarding IoT
Gatouillat et al. [57]	2018	✓	✘	✘	✘	✓	Discussed existing architectures of medical IoT
Shah et al. [58]	2018	✓	✘	✘	✘	✓	Reviews different articles regarding the emergence of IoT and Al applications in healthcare
Qi et al. [59]	2018	✘	✘	✘	✘	✓	Examined the ability of PARM for healthcare IoT
Seneviratne et al. [60]	2017	✘	✓	✘	✘	✓	Discusses the use and challenges of wearable devices
Pawar et al. [61]	2016	✘	✘	✘	✘	✓	Discusses various healthcare IoT applications, challenges regarding security, and counter measures
Ida et al. [62]	2016	✘	✘	✘	✘	✓	Discusses eHealth and clouds contained within healthcare IoT

**TABLE 2.** Applications of RFID by country.

Healthcare Applications of RKII) by Country	
Applications	Country
1. Patient Care 2. Quality of Life 3. Medicine 4. Assets	USA
1. Patient Care 2. Assets	Germany
1. Patient Care 2. Personnel Support	Taiwan
1. Patient Care 2. Quality of Life 3. Medicine	Switzerland
1. Patient Care 2. Quality of Life Medicine	Canada
1.Patient Well-being 2. Quality of Care 3. Medicine	Czech Republic
1. Quality Care	India
Patient Care	Italy
1. Patient Care Supply Management	Netherlands
1. Patient Safety 2. Supply Management	UK

**TABLE 3.** A summary of various architectures and their benefits with respect to medical IoT.

Proposed Architecture	Area(s) of Focus	Benefits)
Edge Computing Architecture for Medical IoT	Home-monitoring	1. Efficiency 2. High-speed transmission
MeDIC	Interoperability	1. Reduced network traffic
Blockchain Based Smart Contracts	Information validation and decision making	1. Higher security 2. Efficient data management
Security-oriented Design Framework for Medical IoT Devices	Security	1. Highly adaptable 2. Consistent security validation 3. Considers embedded system design constraints
Bluetooth-Based Architecture for Contact Tracing	Contact tracing in healthcare facilities	1. Privacy
FC-IoMT architecture	Energy efficiency	1. Reduced energy consumption
LoRa-based Medical IoT architecture	Homecare and hospital services	1. Low complexity

**TABLE 4.** A summary of different standards with their advantages and drawbacks.

Standard	Advantage(s)	Disadvantage(s)
IEEE 802.15.6	1. Handle heterogenous network traffic 2. Provides high QoS 3. Low power consumption	1. Data flow 2. Security
IEEE 802.15.4	1. Low power consumption 2. Low cost 3. Energy efficiency	1. Highly prone to various security attacks
FHIR	1. Multiple implementation libraries 2. Interoperability 3. Easily understood specifications	1. Not backwards-compatible
OpenEHR	1. Global standardization 2. High scalability 3. Designed for clinicians 4. Promotes open sharing of data	1. Complexity
ISO 13485:2016	1. High credibility and prestige 2. Expands potential market of medical IoT 3. Strong focus on quality	1. Cost
ISO/IEEE 11073 PHD	1. Lighter and cheaper communication 2. Simplicity 3. Interoperability	1. Small computing power 2. Limited applicability for medical IoT 3. No standardized output format

**TABLE 5.** Components for Alzheimer's disease framework [168].

Component	Factors
**Sensors**	Vicinity sensing
**Time Intervals**	30 seconds
**A Cluster**	A few sensors per location
**Aggregate**	Determine the location
**Mass of sensors**	Depends on location setup
**Channel**	WiFi Networks of sensor technology
**Utility**	Remote monitoring software
**Deciding Factor**	Patient well-being

**TABLE 6.** Smart hospitals in various countries.

Healthcare Facility	Location	Task(s)

Colchester General Hospital	UK	Well-being

Aventura Hospital	United States	Medicine
Northshore University Hospital		Surveillance

Hospitals in Chennai	South Asia	Robotics

AHMU	Asia	Smart Hospital

**TABLE 7.** Table of biomedical loT devices for monitoring purposes.

Devices	Cost	Year Invented	Weight/Size	Complexity	Genetic/Non-genetic
Oxylink^™^ Remote Oxygen Monitor	$118.00	2019	Ring Design	Real-time	Genetic & Non-genetic
Inner Balance^™^ Heart Math Heart Monitor	$159.00	2017	0.92 Ounces	Fragile	Genetic & Non-genetic
Wellue 02 Ring Wearable Sleep Monitor for Sleep Apnea	$179.99	2020	4.16 Ounces	Real-time	Genetic & Non-genetic
Infrared Forehead Body Temperature Sensor	$39.79	2020	4.6 ounces	Accuracy/Battery Plates	Non-genetic
BACtrack S80 Breathalyzer	$129.99	2004	5.6 Ounces	Recalibration Fails	Genetic/Non-genetic

**TABLE 8.** Table of biomedical IoT devices for prevention.

Devices	Cost	Year Invented	Weight/Size	Complexity	Genetic/Non-genetic
Floor Mat Exit Alarm Elderly Fall Prevention & Anti Wandering Economy System	$139.95	2021	24" × 48"	Battery-powered	Non-genetic
